# Involvement of the Cellular Phosphatase DUSP1 in Vaccinia Virus Infection

**DOI:** 10.1371/journal.ppat.1003719

**Published:** 2013-11-14

**Authors:** Ana Cáceres, Beatriz Perdiguero, Carmen E. Gómez, Maria Victoria Cepeda, Carme Caelles, Carlos Oscar Sorzano, Mariano Esteban

**Affiliations:** 1 Department of Molecular and Cellular Biology, National Centre of Biotechnology, Consejo Superior de Investigaciones Científicas (CSIC), Madrid, Spain; 2 Department of Biochemistry and Molecular Biology, School of Pharmacy, University of Barcelona, Barcelona, Spain; 3 Biocomputing Unit, Centro Nacional de Biotecnología, Consejo Superior de Investigaciones Científicas (CSIC), Madrid, Spain; University of Alberta, Canada

## Abstract

Poxviruses encode a large variety of proteins that mimic, block or enhance host cell signaling pathways on their own benefit. It has been reported that mitogen-activated protein kinases (MAPKs) are specifically upregulated during vaccinia virus (VACV) infection. Here, we have evaluated the role of the MAPK negative regulator dual specificity phosphatase 1 (DUSP1) in the infection of VACV. We demonstrated that DUSP1 expression is enhanced upon infection with the replicative WR virus and with the attenuated VACV viruses MVA and NYVAC. This upregulation is dependent on early viral gene expression. In the absence of DUSP1 in cultured cells, there is an increased activation of its molecular targets JNK and ERK and an enhanced WR replication. Moreover, DUSP1 knock-out (KO) mice are more susceptible to WR infection as a result of enhanced virus replication in the lungs. Significantly, MVA, which is known to produce non-permissive infections in most mammalian cell lines, is able to grow in DUSP1 KO immortalized murine embryo fibroblasts (MEFs). By confocal and electron microscopy assays, we showed that in the absence of DUSP1 MVA morphogenesis is similar as in permissive cell lines and demonstrated that DUSP1 is involved at the stage of transition between IVN and MV in VACV morphogenesis. In addition, we have observed that the secretion of pro-inflammatory cytokines at early times post-infection in KO mice infected with MVA and NYVAC is increased and that the adaptive immune response is enhanced in comparison with WT-infected mice. Altogether, these findings reveal that DUSP1 is involved in the replication and host range of VACV and in the regulation of host immune responses through the modulation of MAPKs. Thus, in this study we demonstrate that DUSP1 is actively involved in the antiviral host defense mechanism against a poxvirus infection.

## Author Summary

Phosphorylation is a post-translational modification that is highly conserved throughout the animal kingdom. Viruses have evolved to acquire their own kinases and phosphatases and to be able to modulate host phosphorylation mechanisms on their benefit. DUSP1 is an early induced gene that belongs to the superfamily of Dual-specificity phosphatases and provides an essential negative feedback regulation of MAPKs. DUSP1 is involved in innate and adaptive immune responses against different bacteria and parasites infections. The use of Knock-out technology has allowed us to understand the role of DUSP1 in the context of VACV infection both in cultured cells and in the *in vivo* mouse model. Here, we have showed that DUSP1 expression is upregulated during VACV infection and that DUSP1 plays an important role in VACV replication. Interestingly, we have demonstrated that the VACV attenuated virus MVA is able to grow in immortalized murine embryo fibroblasts in the absence of DUSP1. *In vivo* results showed that VACV replication-competent WR pathogenesis is enhanced in the absence of DUSP1. Furthermore, we have demonstrated that DUSP1 is involved in the host innate and adaptive responses against VACV. Altogether, we have presented a novel role for DUSP1 in VACV replication and anti-VACV host immune response.

## Introduction

Poxviruses have evolved to efficiently counteract host immune responses through the modulation of extracellular and intracellular environment of the infected cell. Thereby, the ability of a poxvirus to produce a permissive infection in a specific cell type depends on the antiviral mechanisms that the virus is able to block once it infects the cell. VACV is the prototype member of the Poxvirus family, whose large genome of about 200 Kbp encodes a wide array of proteins involved in the control of apoptosis, differentiation, host range, host immune responses and stress-induced signaling pathways [1], [2], [3], [4], [5], [6], [7].

Protein phosphorylation is a conserved mechanism by which the activity of numerous proteins involved in different biological processes is switched on or off [8]. Transient activation of extracellular-regulated kinase (ERK) and jun-kinase (JNK) MAPKs is an essential step in the pathways involved in cellular survival. Therefore, several viruses have evolved to develop strategies that consist of the modulation of these proteins [9], [10], [11]. A specific modulation of cell signaling pathways by VACV is crucial to achieve a productive viral outcome and an efficient viral spread [12], [13]. Hence, VACV not only encodes its own kinases and phosphatases [14], [15], [16] but it is also able to benefit from the activity of cellular proteins such as the ones belonging to the family of MAPKs which consists of p38MAPK, ERK and JNK. For instance, it has been reported that VACV requires ERK activation for an efficient viral replication [17]. MAPKs are involved in important cellular pathways by regulating both physiological and pathological responses that include proliferation, differentiation, stress responses, inflammation, growth arrest and apoptosis [18], [19], [20]. Since MAPKs suffer a context-dependent regulation, a tight control of the magnitude and duration of MAPK activation as well as their subcellular localization are essential for a balanced MAPK signaling [21]. Therefore, as MAPKs are activated by phosphorylation by upstream MAPKKs [22], the cell encodes phosphatases that specifically bind to MAPKs and inactivate them by removing the phosphates on the Thr-Xaa-Tyr motif. Among these phosphatases, DUSPs provide an important negative feedback mechanism for MAPK activation [23]. The family of DUSPs shares a common kinase interaction motif (KIM) located at the N-terminal region and a catalytic domain at the C-terminal region.

DUSP1, as the archetypal member of the family, has been extensively studied. It is encoded by an immediate-early gene that was first discovered in 1985 as one of the genes expressed in cultured murine cells during the G_0_/G_1_ transition [24]. Since DUSP1 knock-out (KO) mice were healthy and presented no obvious phenotype related to MAPK activity [25], it was not until the last decade that DUSP1 grew in importance due to the novel phenotype of DUSP1 KO mice found after treatment with corticoids. DUSP1 is ubiquitously expressed in the organism and it is known to bind p38MAPK, JNK or ERK depending on the cell status [26], [27]. DUSP1 expression is strongly upregulated upon several stimuli such as oxidative stress, hypoxia, growth factors, glucocorticoids, heat shock and UV [28], [29], [30], [31], [32]. DUSP1 is tightly regulated at transcriptional, translational and post-translational levels. Some well-characterized examples are the phosphorylation of Serines 359 and 364, which enhances the half-life of DUSP1 [33] or the acetylation of DUSP1 that mediates important events on the host inflammatory response [34], [35]. As an important MAPK regulator, DUSP1 is involved in a wide variety of cellular processes such as obesity, transplantation, cancer, depressive behavior and inflammation [36], [37], [38], [39], [40].

By microarray analysis of VACV-infected cells, we [41], [42] and others [43], [44] have identified DUSP1 as one of the genes specifically induced by VACV infection. Although several reports have highlighted the importance of DUSP1 in parasite and bacterial diseases [45], [46], [47], there is limited information on the involvement of DUSP1 in viral infections. The present study is focused on the role of DUSP1 in VACV infection and more specifically in the replication of wild-type (WT) VACV Western Reserve (WR) and VACV attenuated viruses Modified Vaccinia virus Ankara (MVA) and NYVAC. We have addressed this issue both *in vitro*, by using cultured DUSP1 WT and KO cells, siRNA technology and nucleofection, and *in vivo* through a DUSP1 KO mouse model. We showed that replication of WR and MVA is enhanced in the absence of DUSP1 and that, interestingly, DUSP1 is a key factor during MVA morphogenesis in murine cells. Moreover, we demonstrated the influence of DUSP1 in host innate and adaptive immune responses elicited by VACV. Overall, the present study reveals a novel role for DUSP1 in VACV infection and provides new insights into the still not fully understood MVA host restriction process.

## Materials and Methods

### Ethics statement

Animal experimental protocols were approved by the Ethical Committee of Animal Experimentation (CEEA-CNB) of Centro Nacional de Biotecnologia (CNB-CSIC, Madrid, Spain) in strict accordance with Spanish national Royal Decree (RD 1201/2005) and international EU guidelines 2010/63/UE about protection of animals used for experimentation and other scientific purposes and Spanish national law 32/2007 about animal welfare in their exploitation, transport and sacrifice and also in accordance with the Royal Decree (RD 1201/2005). Permit numbers: 10-018 and 10-023.

### Cells and viruses

African green monkey kidney cells (BSC40) from ATCC were grown in Dulbecco's Modified Eagle's medium (DMEM) with penicillin (100 U/ml, Invitrogen), streptomycin (100 µg/ml, Invitrogen), L-glutamine (2 mM; Merck) and supplemented with 10% newborn calf serum (NCS; Sigma). Human HeLa cells (human cervix adenocarcinoma cell line), baby hamster kidney cells (BHK-21), DF-1 (a spontaneously immortalized chicken embryo fibroblast cell line), all from ATCC, and DUSP1 wild-type and knock-out murine embryonic fibroblasts were cultured in complete DMEM supplemented with 10% fetal calf serum (FCS; Sigma). Cells were maintained in a humidified air-5% CO_2_ atmosphere at 37°C or 39°C (for DF-1 cell line). VACV wild-type Western Reserve (WR) was grown in monkey BSC40 cells and purified by sucrose gradient banding. NYVAC (kindly provided by Sanofi-Pasteur) and MVA (kindly provided by G. Sutter) were grown in BSC40 cells and in BHK-21 cells, respectively, and purified by two subsequent 36% sucrose (w/v) cushions. After all infections, complete DMEM supplemented with 2% NCS or FCS was added to the cultured cells. WR, MVA and NYVAC were titrated in DF-1 cells by immunostaining plaque assay as previously described [48]. Where indicated, purified WR was inactivated by ultraviolet light during 20 min [49].

### Quantitative Real-Time RT-PCR

HeLa cells were mock-infected or infected with WR, MVA or NYVAC at 5 PFU/cell. Where indicated, cells were infected in the presence of Cycloheximide (CHX) at a final concentration of 100 µg/µl. Total RNA was isolated using RNeasy Mini kit (Qiagen) following manufacturer's instructions. Total RNA (1 µg) was digested with DNases to avoid genomic DNA contamination. Reverse-transcription PCR (RT-PCR) was performed using Superscript first-strand synthesis system (Invitrogen). 50 ng of cDNA was assayed for human DUSP1 expression using specific Taqman probes (Applied Byiosystems) and TaqMan Universal PCR MasterMix (No AmpErase UNG). Duplicates were assayed for each sample. Data were acquired with an ABI PRISM 7000 sequence detection system and analyzed with ABI PRISM 7000 SDS version 1.2.3 software (Applied Biosystems).

### Protein analysis by immunoblotting

Cells were infected with WR, MVA or NYVAC at 5 PFU/cell, harvested at different times post-infection and protein extracts obtained using lysis buffer following manufacturer's recommendations (Cell lysis Buffer, Cell Signaling). Equal amounts of protein (50 µg) were fractionated by 10% SDS-polyacrylamide gel electrophoresis (SDS-PAGE) and then transferred to nitrocellulose membrane (Bio-Rad Laboratories) in a wet blotting apparatus (Bio-Rad) for 50 min. Membranes were blocked with TBS-Tween 20 0,1% BSA 5% and then incubated with specific antibodies against viral proteins such as E3 (kindly provided by B. Jacobs), A10 and L4R (kindly provided by D. Hruby), A14 (CNB, CSIC), A17 (CNB, CSIC), A17-N (kindly provided by J. Locker) and A17-C (CNB, CSIC) or cellular proteins such as α-tubulin, P-DUSP1, P-ERK1/2 and P-JNK (Cell Signaling) or total DUSP1 (Santa Cruz Biotechnology), ERK1/2 or JNK (Cell Signaling). The anti-rabbit-HRPO (Sigma) was used as secondary antibody and the immunocomplexes were detected by enhanced chemiluminescence (ECL, GE Healthcare). The viral DNA synthesis inhibitor Arabinofuranosyl Cytidine (Ara-C) and protein synthesis inhibitor CHX from (SIGMA) were added to cell culture media at 40 µg/ml and 100 µg/ml, respectively.

### Analysis of viral growth

To determine viral growth profiles, duplicate samples of monolayers of MEFs grown in 12-well tissue culture plates were infected at 0.01 PFU/cell with WR, MVA or NYVAC. At 0, 24 and 48 hours post-infection, samples were collected and titrated as described previously [6]. Specific MAPKs inhibitors, ERK inhibitor UO126 (Cell Signaling) and p38MAPK inhibitor SB203580 (Calbiochem), were added to the cell culture at a concentration of 10 or 50 µM and 5 µM, respectively, for 30 min or 1–8 h (UO126) or 30 min (SB203580) before the infection.

Alternatively to KO cells, DUSP1 expression was suppressed with interference RNAs. Duplicate samples of subconfluent monolayers of HeLa cells grown in 12-well tissue culture plates were treated with DUSP1 specific siRNAs (siGENOME SMARTpool, Dharmacon; target sequence 1: CCAUUGUCCCAACCAUUU; target sequence 2: CAACGAGGCCAUUGACUUC; target sequence 3: CCACCACCGUGUUCAACUUÇ; target sequence 4: GCAUAACUGCCUUGAUCAA) or Scramble siRNA (Applied Biosystems) as a control, at a final concentration of 50 nM using Lipofectamine 2000 (Invitrogen). After 48 hours of incubation with the siRNAs, cells were infected with WR or MVA at 0.1 PFU/cell. At 0, 24 and 48 hours post-infection cells in medium were collected and virus titrated as previously described [6].

### Nucleofection assay

DUSP1 KO MEFs were nucleofected using 4D-Nucleofector (Lonza) and Amaxa P3 Primary Cell kit (Lonza) following manufacturer's instructions. Briefly, 2×10^6^ cells were resuspended in 100 µl P3 Buffer Mix and nucleofected with 6 µg of pSG5-DUSP1 plasmid (kindly provided by E. Perdiguero) or pSG5-empty plasmid as a control. After 24 hours of incubation, MEFs were infected with WR or MVA at 0.1 PFU/cell and viral titers were determined at 0, 24 and 48 hpi by immunoplaque assay in DF-1 cells.

### Immunofluorescence

MEFs were infected with WR, MVA or NYVAC at 5 PFU/cell and at 5.5 or 16 hpi. Cells were fixed with 4% paraformaldehyde (PFA) and permeabilized with 0.05% saponin in 2% FCS-supplemented phosphate-buffered saline (PBS). Specific antibodies against viral proteins A27 (1∶2000), A17 and A14 (1∶1.000) were used (CNB). Bound primary antibodies were detected with AlexaFluor-488 or −594 conjugated antibodies specific for mouse or rabbit (Invitrogen 1∶500). β-Actin was detected with a TRITC-conjugated probe anti-Phalloidin (Sigma, 1∶500). Cell nucleus was stained with 4′, 6-diamino-2-phenylindole (DAPI; Sigma, 1∶200). To block DUSP1 in WT cells, siRNA treatment was performed on subconfluent monolayers of MEFs using Lipofectamine 2000 (Invitrogen) as transfection agent and a mix of two DUSP1 specific siRNAs (Applied Biosystems) at a final concentration of 100 nM. After 24 hours of incubation, cells were infected with VACV and specific viral proteins were detected by immunofluorescence and confocal microscopy as described above.

### Electron microscopy

Confluent monolayers of MEFs were mock-infected or infected with MVA at 5 PFU/cell and at 16 hpi cells were fixed for 2 hours with 4% PFA+2% glutaraldehyde in NaH_2_PO_4_/Na_2_HPO_4_ 0.1M pH 7.4 buffer. Samples were then washed (3 times, 10 min each) with NaH_2_PO_4_ pH 7.4 buffer, scrapped and harvested in 1.5 ml of the same buffer. Fixed samples were processed by conventional embedding in the epoxi-resin EM-812 (Taab Laboratories, Adermaston, Berkshire, UK). Ultrathin sections of 70 nm thick were collected on formvar-coated parallel-bar copper grids and analyzed with a JEOL 1011 transmission electron microscope. Quantitative analysis of viral forms by electron microscopy was performed analyzing 30 infected cells per cell line (DUSP1 WT and KO cells). The results represent the percentage of each viral intermediate in the total of viral particles quantified.

### Mice, viral inoculation, pathogenicity analysis and sample collection

DUSP1 KO mice were generated by Jackson laboratories using embryos with a 129S2/SvPas genetic background. Jackson Labs provided Dr. Caelles lab with the resulting mice and they were backcrossed in a C57BL/6 background for at least 11 mice generations. MEFs from wild type and KO mice were generated independently from mouse embryos by Caelle's and Esteban's labs following standard procedures.

Groups of 8–10 week-old DUSP1 WT or KO mice were intranasally (i.n., n = 5, 20 µl inoculum), intraperitoneally (i.p., n = 3–4, 200 µl inoculum) inoculated or skin scarified (s.s., n = 3–4, 10 µl inoculum) with different challenge doses of WR (5×10^5^–5×10^6^ PFU/mouse), MVA or NYVAC (5×10^6^–2×10^7^ PFU/mouse). Representative examples of three independent experiments are shown in the figures. Weight loss, mortality and signs of illness, such as reduced mobility and ruffled fur, were daily evaluated using a 0–3 scale (healthy-ill). For *in vivo* viral replication assays, mice were sacrificed at various times post-inoculation and spleen, liver, ovaries (i.p.), lungs (i.n.) or tail tissue (s.s.) were excised, washed with sterile PBS and stored at −70°C. Tissue extracts were obtained by homogenizing representative sections of each tissue. Homogenized samples were subjected to three cycles of freezing and thawing, sonicated and centrifuged (10 min/2000 rpm); supernatants were collected and titrated by immunostaining plaque assay. Serum was obtained by submaximal vein bleedings 3 or 24 hours post-inoculation and was allowed to clot 1 hour at 37°C. After a 4°C overnight incubation, they were spun down in a microcentrifuge, serum was collected, aliquots were prepared, pooled and stored at −20°C.

### Cytokine analysis

Sera from mock-infected or WR, MVA or NYVAC-infected mice were assayed for detection of IL-6, TNF-α, IL-1β and IL-10 using LUMINEX technology according to the manufacturer's instructions. All samples were kept at −20°C until use.

### Measurement of luciferase activity

Recombinant viruses WR-Luc, MVA-Luc and NYVAC-Luc expressing luciferase have been previously described [50], [51]. Samples from mice i.p. inoculated with these viruses at 2×10^7^ PFU/mouse were homogenized in 200–400 µl of luciferase assay reagent (Promega) per sample. Clarified supernatants were used to measure luciferase activity in the presence of luciferine and ATP according to the manufacturer's instructions, using Lumit LB 9501 luminometer (Berthold). The luciferase activity is represented as luciferase units per miligram of protein (U/mg).

### Intracellular cytokine staining (ICS)

The specific VACV-induced T cell response was analyzed by ICS and flow cytometry fluorescence-activated cell sorting analysis (FACS). After isolation of splenocytes from infected mice, samples were resuspended in RPMI 1640 supplemented with 10% FCS and containing 1 µg/ml Golgiplug (BD Biosciences) to inhibit cytokine secretion. Cells were stimulated with E3 peptide (sequence: VGPSNSPTF, CNB, CSIC) added to the cells at a final concentration of 5 µg/ml. After 6 hours of incubation at 37°C and 5% CO_2_, cells were washed, fixed, permeabilized, using the BD Cytofix/Cytoperm Kit (Becton Dickinson), and stained intracellularly using the appropriate fluorochrome-conjugated antibodies. To analyze the adaptive immune responses, the following fluorochrome-conjugated antibodies were used: CD3-FITC or -PerCP, CD4-Alexa 700 or –APCCy7, CD8-PerCP or –V500, IL-2-PE or –PE-Cy7, IFN-γ-APC or –PE-Cy7 and TNF-α-PE-Cy7 or -PE. All antibodies were from BD Biosciences. Cells were acquired using an LSRII flow cytometer (Becton Dickinson). Dead cells were excluded using the violet LIVE/DEAD stain kit (Invitrogen). Lymphocytes were gated on a forward scatter area versus side scatter area pseudo-color dot plot. To analyze the adaptive immune responses, CD4^+^ and CD8^+^ T cells (previously gated on CD3^+^ cells) were gated versus IFN-γ, TNF-α and IL-2 and then combined together using the boolean operator. Sample analysis was performed using FlowJo version 8.5.3 (Tree Star, Ashland, OR).

### Data analysis and statistics

For the statistical analysis of the viral growth and the differences between groups of mice a Student T test was performed. Statistical significance is represented as p<0.05 (*), p<0.005 (**) or p<0.001 (***).

For the statistical analysis of ICS data, we used a novel approach that corrects measurements for the medium response (RPMI) and calculates confidence intervals and *p* values of hypothesis tests [52], [53]. Only antigen responses values significantly higher than the corresponding RPMI are represented and the background for the different cytokines in the unstimulated controls never exceeded 0.05%. We used the data analysis program Simplified Presentation of Incredibly Complex Evaluations (SPICE, version 4.1.5, Mario Roederer, Vaccine Research Center, NIAID, NIH) to analyze and generate graphical representations of T cell responses detected by polychromatic flow cytometry. All values used for analyzing proportionate representation of responses are background-subtracted.

### Accession numbers

- Tubulin: human, P68366, UniprotKB.

- DUSP1: human, P28562, UniprotKB.

- DUSP1: mouse, P2563, UniprotKB.

- ERK1: mouse, Q63844, UniprotKB.

- ERK2: mouse, P63085, UniprotKB.

- JNK1: mouse, Q01Y86, UniprotKB.

- JNK2: mouse, Q9WTU6, UniprotKB.

- p38MAPK: mouse, P47811, UniprotKB.

- IFN-γ: mouse, P01580, UniprotKB.

- IL2: mouse, P04351, UniprotKB.

- TNF-α: mouse, P06804, UniprotKB.

- IL6: mouse, P08505, UniprotKB.

- IL1β: mouse, P10749, UniprotKB.

- IL10: mouse, P18893, UniprotB.

## Results

### DUSP1 expression is upregulated upon VACV infection

**Figure 1:**
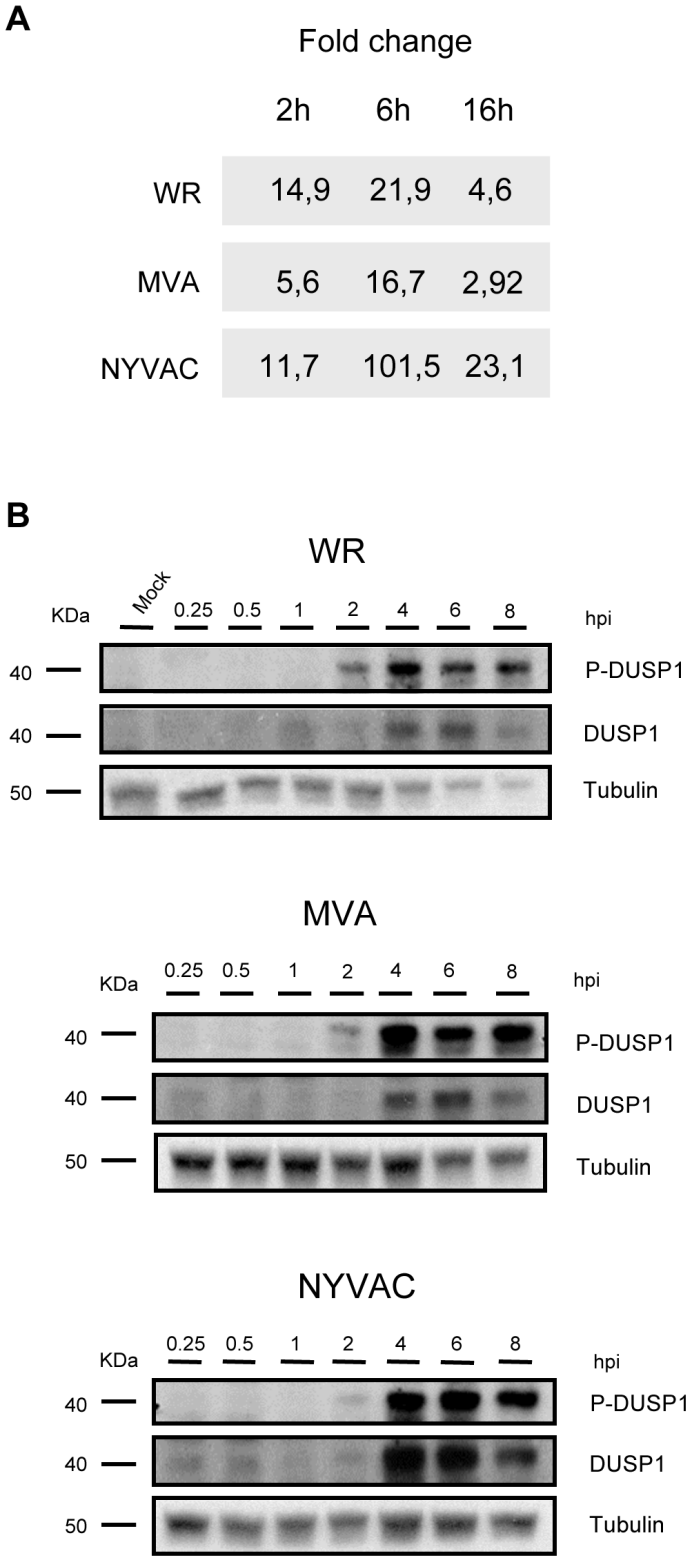
DUSP1 expression is upregulated upon VACV infection. **A**) DUSP1 mRNA levels after VACV infection. HeLa cells were infected with WR, MVA or NYVAC at a MOI of 5 and samples from 2, 6 and 16 hours post-infection were assayed by RT-PCR. **B**) DUSP1 protein levels after VACV infection. HeLa cells were mock-infected or infected as in A) and equal amounts of protein from cell extracts were analyzed by Western Blot using specific antibodies against DUSP1, P-DUSP1 or tubulin.

We and others have previously described using microarray technology that DUSP1 mRNA transcription is specifically upregulated during VACV infection [42], [43], [44], [42]. To validate these previous observations, we carried out RT-PCR and Western-blot analyses from HeLa cells infected with the virulent WR strain or with the attenuated VACV mutants MVA and NYVAC. As shown by RT-PCR, in HeLa cells infected with WR, MVA or NYVAC, there was an increase in DUSP1 mRNA levels at the different times post-infection analyzed (Fig. 1A). The induction was more pronounced at 6 hpi, especially for NYVAC infection. The increase in DUSP1 protein expression levels following VACV infection was confirmed by Western-blot analysis (Fig. 1B). DUSP1 protein was upregulated during the infection with each one of the three viruses used and this increase peaked between 4 and 6 hpi. The highest levels of DUSP1 induction were observed upon NYVAC infection where an increase in protein expression was still detected at 8 hpi. This upregulation was VACV-specific since in uninfected control cells DUSP1 protein level was below the limit of WB detection.

To determine if VACV not only triggers enhanced protein synthesis but also affects protein stability, we used an antibody that recognizes specifically phosphorylated serines 359 and 364, which are the residues responsible for DUSP1 stabilization [33]. We observed phosphorylation of DUSP1 after the infection with each one of the three viruses used. The highest phosphorylation levels of DUSP1 were observed at 4 hpi (Fig. 1B). The findings of Fig. 1 showed that DUSP1 mRNA transcription and DUSP1 protein expression as well as DUSP1 phosphorylation were specifically modulated by VACV infection in HeLa cells.

### DUSP1 protein induction and phosphorylation require early VACV protein synthesis and ERK activation

**Figure 2:**
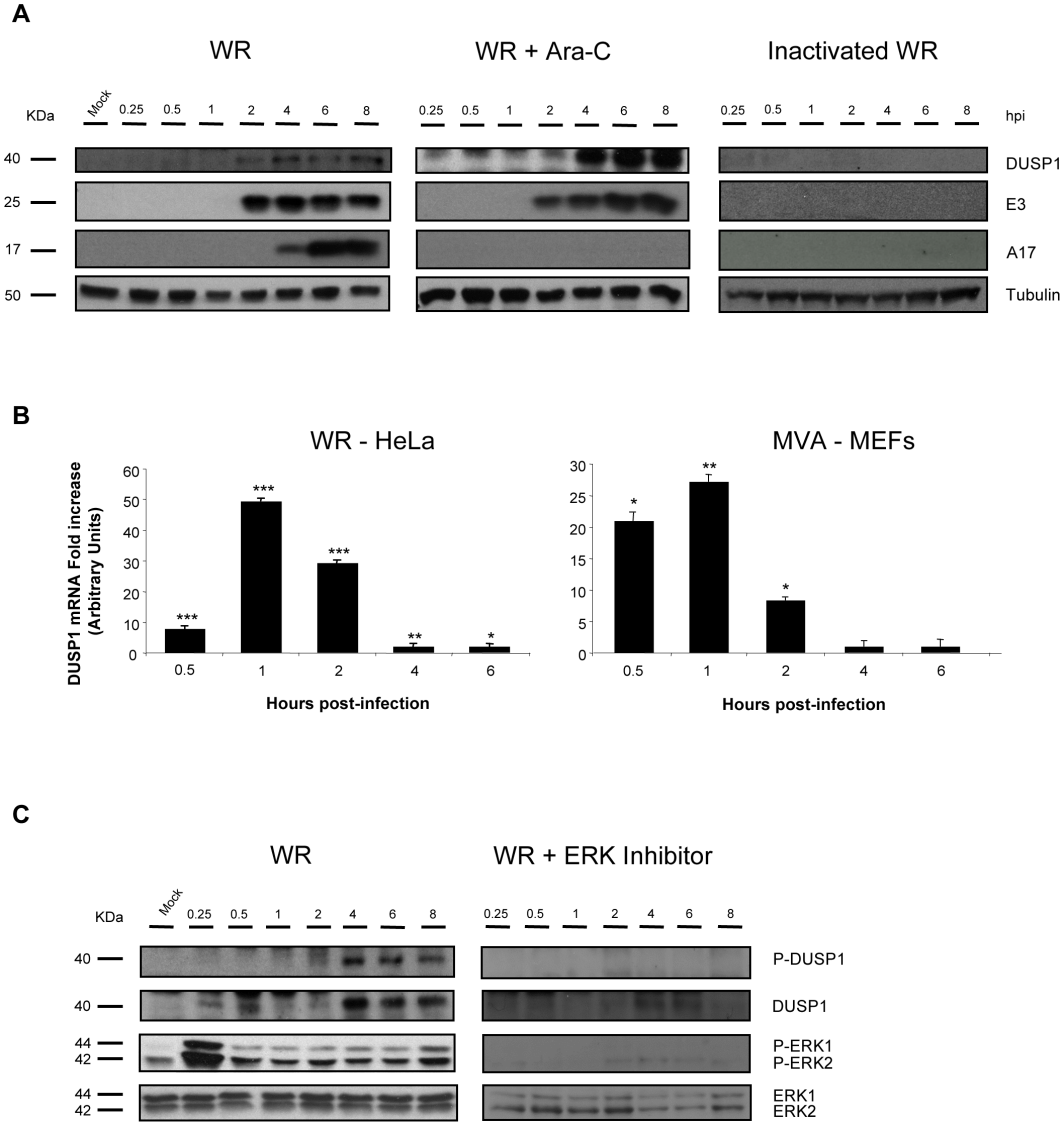
DUSP1 induction and phosphorylation requires early VACV gene expression and ERK activation. **A**) DUSP1 protein levels after WR infection. HeLa cells with were treated with Ara-C (40 µg/ml) or left untreated and then subsequently mock-infected, infected with UV-inactivated WR or infected with WR at 5 PFU/cell. Equal amounts of proteins were analyzed by Western-blot using specific antibodies against DUSP1 or tubulin or viral proteins such as E3, A4 and A17. Lower graph represents fold increase of DUSP1 mRNA of CHX-treated WR-infected HeLa cells versus untreated WR-infected cells. mRNA levels are represented in Arbitrary Units. **B**) DUSP1 mRNA levels after VACV infection. HeLa cells or DUSP1 WT MEFs were treated with CHX (100 µg/ml) or left untreated and then subsequently infected at 5 PFU/cell with WR or MVA, respectively. Graphs represent fold increase of DUSP1 mRNA of CHX-treated infected cells versus untreated infected cells. mRNA levels determined by qRT-PCR are represented in Arbitrary Units. **C**) DUSP1 phosphorylation after WR infection. HeLa cells were treated with ERK inhibitor UO126 (50 µM/8 h) or left untreated and the infected as in A). Cells extracts were analyzed by Western-blot using antibodies against DUSP1, P-DUSP1, ERK, P-ERK or tubulin. p<0.05 (*), p<0.005 (**) and p<0.001 (***).

To define the role of viral proteins on DUSP1 induction, we treated HeLa cells with a UV-inactivated WR virus which is unable to synthesize viral RNA or protein or with an inhibitor of viral DNA synthesis, Arabinofuranosyl Cytidine (Ara-C). In the presence of Ara-C (Fig. 2A, central panel), early VACV proteins, such as E3 (p25), were expressed, however, late viral proteins such as A17 (p17) were not produced. In Ara-C-treated samples, DUSP1 expression showed the same kinetics as observed in WR-infected cells (Fig. 2A, left panel), whereas DUSP1 induction was not observed in UV-inactivated WR-infected cells (Fig. 2A, right panel) nor with a 55°C inactivated WR (not shown). These findings reveal that early but not late viral gene expression is required for VACV-mediated DUSP1 protein upregulation.

To define which viral component is responsible for the induction of DUSP1 mRNA, we infected HeLa cells with WR in the presence of CHX, which allows viral mRNA but not protein synthesis, and determined the levels of DUSP1 mRNA by quantitative RT-PCR. As shown in Fig. 2B (left panel), in the presence of CHX there was an increase of DUSP1 mRNA levels in comparison with untreated infected samples, indicating that DUSP1 mRNA expression is dependent on viral RNA synthesis. In order to analyze whether this result could also be observed with an attenuated VACV and to test another cell culture model, we infected DUSP1 WT MEFs with MVA and analyzed DUSP1 mRNA. Fig. 2B (right panel) shows for MVA in MEFs similar kinetics for DUSP1 mRNA to HeLa cells.

In order to determine whether during VACV infection DUSP1 stabilization is mediated by ERK phosphorylation or other cellular kinase or if a viral-encoded kinase is involved, we used an ERK inhibitor (UO126). ERK inhibitor was added to the culture medium 30 min before infection and was present in the cell culture at all times during infection. In the presence of ERK inhibitor (Fig. 2C), ERK phosphorylation and, consequently ERK activation, was completely abrogated and we could not detect DUSP1 phosphorylation.

The findings of Figure 2 reveal that induction of DUSP1 mRNA and protein required viral mRNA synthesis and early viral gene expression. Moreover, DUSP1 phosphorylation during VACV infection is mediated by ERK.

### DUSP1 modulates VACV replication in cultured cells

**Figure 3:**
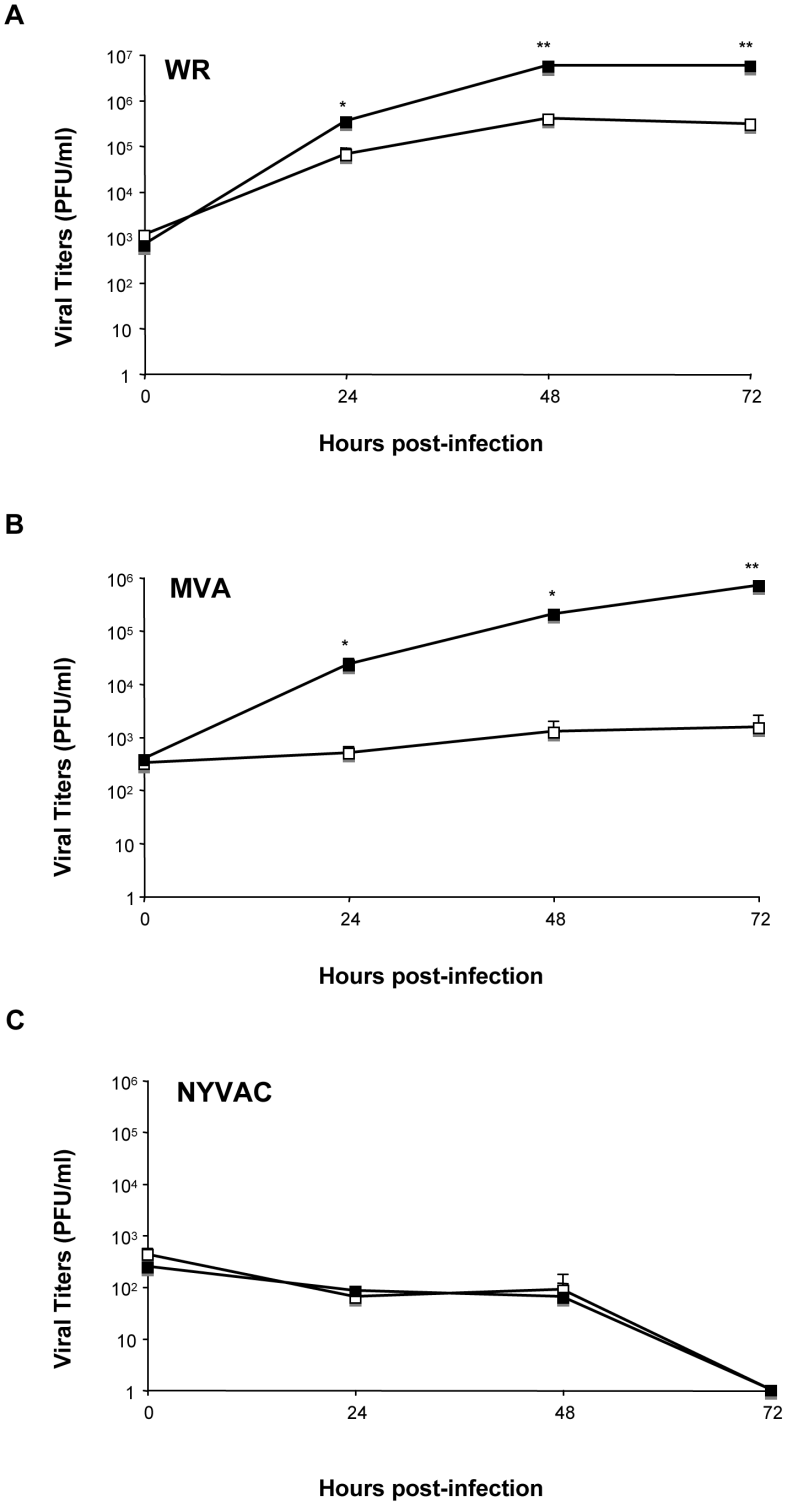
DUSP1 modulates VACV replication in cultured cells. DUSP1 WT and KO MEFs were infected at 0.01/cell with WR (A), MVA (B) or NYVAC (C). Samples were collected at 0, 24, 48 and 72 hours post-infection and viral titers were determined by immunostaining plaque assay in DF1 cell line. One representative example of three independent experiments assayed in duplicate is represented. p<0.05 (*), p<0.005 (**) and p<0.001 (***).

DUSP1 plays an important role in the negative regulation of MAPKs upon different stimuli, including bacteria and parasite infections [54]. Nevertheless, little is known about the function of DUSP1 in viral infections. To assess this issue, we decided to determine the effect of the absence of DUSP1 on the replication of WR, MVA or NYVAC. We observed a significant increase (24 hpi: *p*<0.05; 48 and 72 hpi: *p*<0.005) in WR replication in KO MEFs in comparison with WT MEFs (Fig. 3A). Surprisingly, when we infected the cells with MVA, which also cannot replicate in most mammalian cell lines, we detected higher viral titers in KO MEFs in comparison with the WT MEFs; this significant difference (24 and 48 hpi: *p*<0.05; 72 hpi: *p*<0.005) in replication of about two logs higher was evident at 24 hpi and increased with time (Fig. 3B). Productive MVA infection required DUSP1 KO MEF immortalization process, as MVA produced abortive infections in primary DUSP1 KO MEFs obtained from DUSP1 KO mice (data not shown). However, we could not detect NYVAC replication in KO nor in WT MEFs (Fig. 3C), which was consistent with the fact that NYVAC is unable to replicate in murine cell lines.

**Figure 4:**
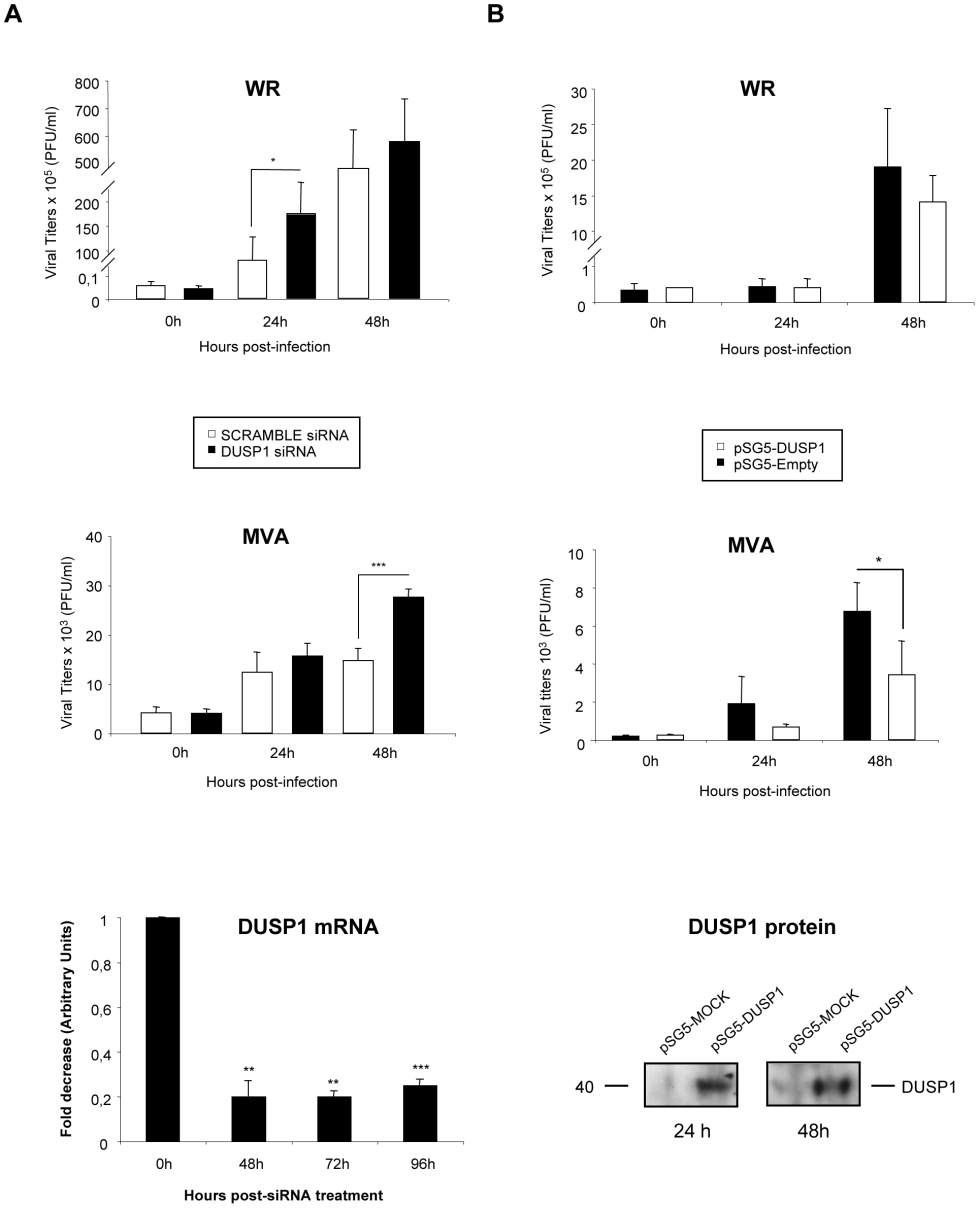
The increased VACV replication in cultured cells is DUSP1 specific. **A**) HeLa cells were treated with 50 nM of either DUSP1 siRNAs or scramble siRNA as a control and after 24 hours of incubation cells were infected with WR or MVA at 0.1 PFU/cell. Samples were collected at 0, 24 and 48 hours and viral titers were determined by immunostaining plaque assay in DF-1 cell line. Lower panel shows fold decrease of DUSP1 mRNA levels in siRNA-treated cells compared to mRNA levels in scramble siRNA-treated cells. **B**) DUSP1 KO MEFs were nucleofected with 6 µg of either pSG5-empty or pSG5-DUSP1 and DUSP1 protein expression analyzed by WB at 24 and 48 hours post-transfection (lower panel). After 24 hours of incubation with siRNAs, cells were infected with WR or MVA at 0.1 PFU/cell. Samples were collected at 0, 24 and 48 hours and viral titers were determined as in A. One representative example of two independent experiments assayed in duplicate is represented. p<0.05 (*), p<0.005 (**) and p<0.001 (***).

In order to further confirm these results, we treated HeLa cells, which is a permissive cell line for WR but host-restricted to MVA replication, with specific siRNA to suppress DUSP1 expression. As shown in Fig. 4A, DUSP1 mRNA expression was efficiently suppressed (lower panel) and viral titers of both WR and MVA were significantly increased (upper and middle panels) (WR, 24 hpi: p<0.05; MVA, 48 hpi: p<0.001) in HeLa cells treated with DUSP1 siRNAs compared to cells treated with a scramble siRNA. This phenotype mimics what it was that previously observed in DUSP1 KO cells infected with either WR or MVA (Fig. 3).

In addition, to further prove the role of DUSP1 in VACV replication, we nucleofected a DNA vector expressing DUSP1 gene into DUSP1 KO MEFS, then cells were infected with WR or MVA and viral titers were determined at 0, 24 and 48 hpi. As shown in Fig. 4B, DUSP1 expression was successfully restored into KO MEFs (WB in lower panel) and viral titers were significantly (MVA, 48 hpi: p<0.05) reduced in cells infected with MVA expressing DUSP1 (middle panel) in comparison with control cells. This effect was not observed in the case of WR infection (upper panel).

The results of Fig. 4 establish that DUSP1 plays a role in the replication of VACV, and this effect is more prevalent in the case of the MVA strain.

### DUSP1 is responsible for the host restriction of MVA in MEFs

**Figure 5:**
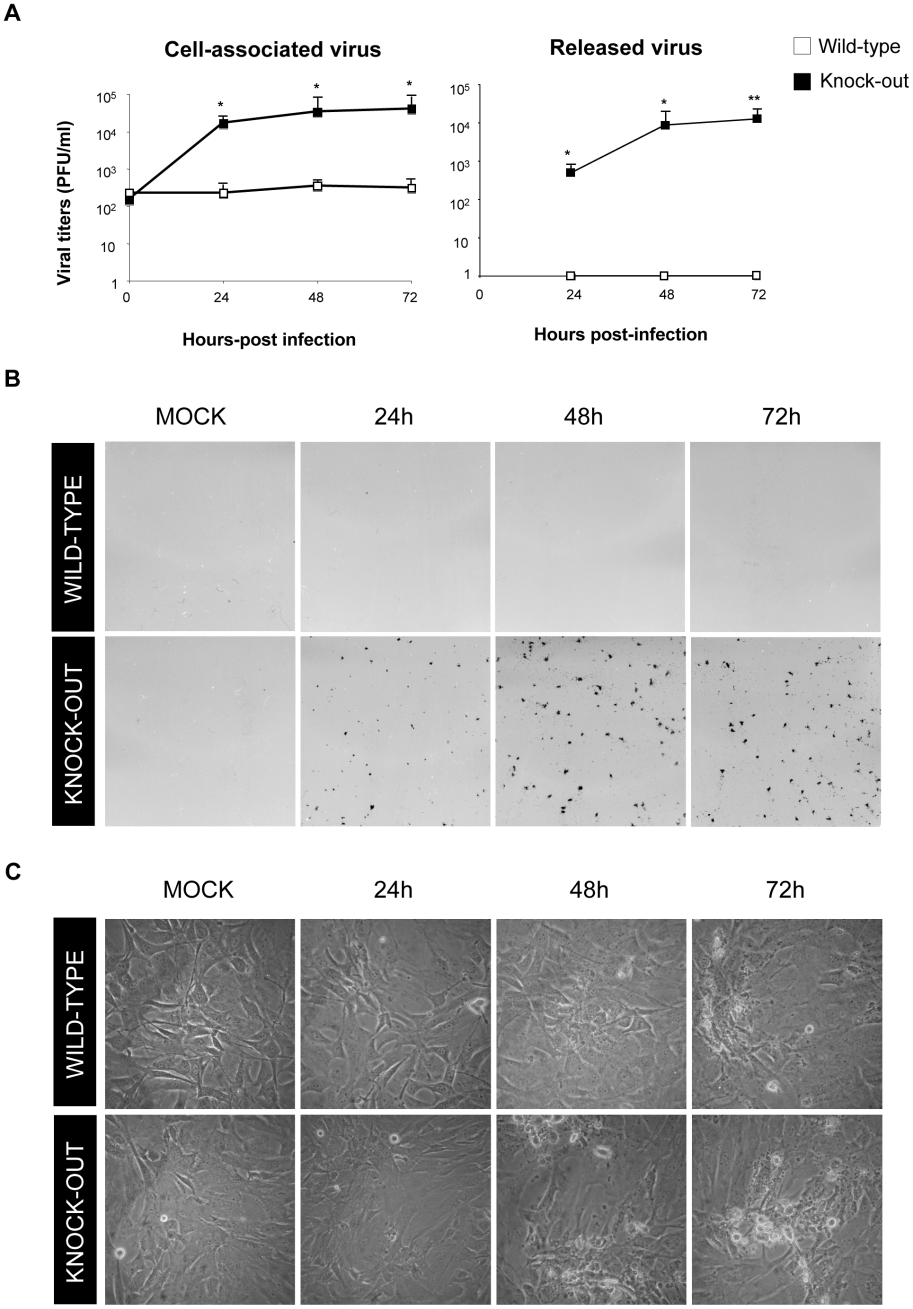
DUSP1 is responsible for the host restriction of MVA in MEFs. **A**) Viral growth of MVA in DUSP1 WT and KO cells infected at 0.01 PFU/cell. Samples of released and cell-associated virus were collected at 0, 24, 48 and 72 hours post-infection and viral titers were determined by immunostaining plaque assay in DF-1 cells. **B**) Immunostaining plaque assay of MVA infection in DUSP1 WT and KO cells infected as in A. **C**) Cytopathic effect observed in DUSP1 WT and KO cells infected with MVA as in A. p<0.05 (*), p<0.005 (**) and p<0.001 (***).

To further characterize the role of DUSP1 in MVA replication in MEFs, we performed a viral growth assay to evaluate the levels of cell-associated virus and released virus from infected cells. The titration of cell-associated virus showed a difference of over 2 logs in MVA production between KO and WT cells (Fig. 5A). This difference was even more evident in the case of released virus since we could not detect the presence of virus in the cell supernatants of WT-infected MEFs. Moreover, when we performed immunoplaque assay of MVA-infected WT or KO MEFs, we could only observe virus plaques in the absence of DUSP1 at any time point analyzed (Fig. 5B). There were differences in the cytopathic effect (CPE) produced by MVA infection between WT and KO cells (Fig. 5C), with a more pronounced CPE in KO MEFs (lower panels) compared to WT MEFs (upper panels). Altogether, these results reveal a distinct behavior of MVA in KO MEFS in comparison with WT MEFs indicating that the host restriction of MVA replication in murine cells is overcome in the absence of DUSP1.

### DUSP1 acts in the transition between immature to mature viral forms of MVA

Considering our previous data showing that MVA was able to complete its viral cycle in MEFs in the absence of DUSP1, we next wanted to determine which of the events blocked during MVA morphogenesis is overcome by the action of DUSP1. To address this issue, we studied early, intermediate and late events during MVA morphogenetic process in the absence of DUSP1.

**Figure 6:**
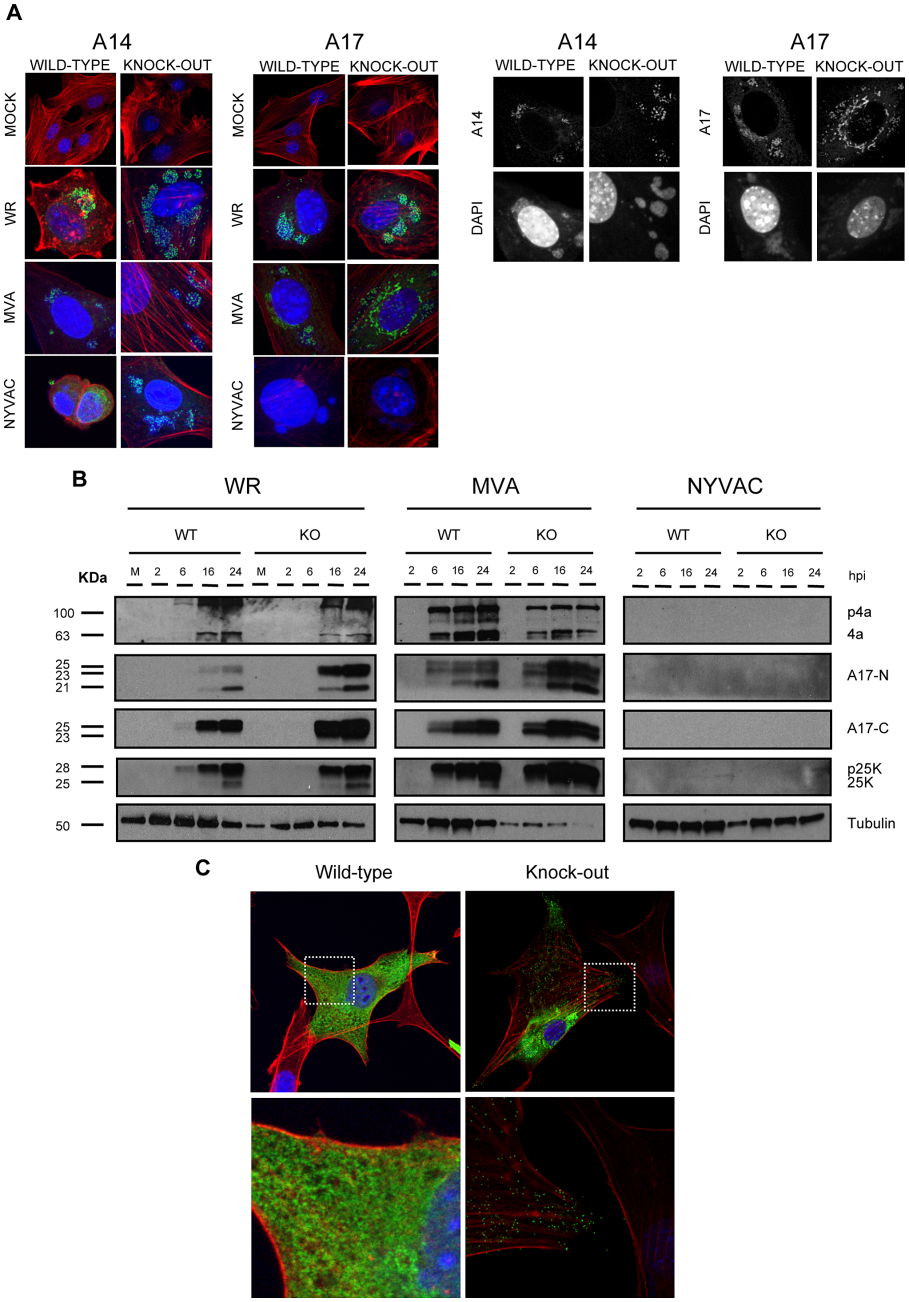
DUSP1 is involved in steps occurring before mature virion formation. **A**) Confocal microscopy of DUSP1 WT and KO cells. Cells were infected at 5 PFU/cell with either WR, MVA or NYVAC. Samples were fixed at 5 h 30 min and stained with antibodies against viral proteins A14 or A17 (green), or probes that detect phalloidin (red) and DNA (DAPI, blue). Black panels show representative images of WT and KO cells infected with MVA. **B**) Western-blot analysis of DUSP1 WT and KO cells infected at 5 PFU/cell with either WR, MVA or NYVAC. Samples were collected at 2, 6, 16 and 24 hours post-infection and analyzed by Western-blot with antibodies against viral proteins A10, L4 and A17 and cellular protein tubulin. **C**) Confocal microscopy of DUSP1 WT and KO cells infected with MVA at 5 PFU/cell and fixed at 16 hpi. Lower panels represent magnifications (5×) of the pictures above. Samples were stained with antibodies against viral protein A27 (green), phalloidin (actin, red) and DNA (DAPI, blue).

First, we analyzed by immunofluorescence and confocal microscopy the expression of the viral membrane proteins A14 (p16) and A17 (p17), which play key roles during the formation of crescents [55], [56]. In DUSP1 WT and KO MEFs infected with WR, MVA or NYVAC at 5 PFU/cell (Fig. 6A), we did not observe differences in the expression pattern of A14 or A17 between WT and KO MEFs. Black panels in Fig. 6A showed a punctuated pattern of both A14 and A17 in MVA-infected cells that localized with viral factories. Since the pattern of localization of A14 and A17 was similar in WT and KO-infected cells, we can suggest that this step of viral morphogenesis is not dependent on DUSP1.

During VACV morphogenesis, the processing of viral membrane and core proteins is required prior to formation of the mature virion. Some of the proteins that need proteolytic cleavage during infection are A10 (p4a), L4 (p25K) and A17 (p17). In order to analyze this event, we infected WT and KO cells with WR, MVA or NYVAC and determined the expression of these three viral proteins at 2, 6, 16 and 24 hours post-infection by Western-blot analysis using antibodies that recognize the processed forms of the proteins: 4a, 25K, A17-C (processed C-terminus) and A17-N (unprocessed N-terminus) (Fig. 6B). In the case of WR and MVA, processed forms of these proteins were found in both WT and KO-infected cells. However, as observed in the right panel, we could not detect any of these proteins in WT or KO cells infected with NYVAC, consistently with previous reports demonstrating that NYVAC does not produce certain late viral proteins in non-permissive cells lines [6]. These findings reveal that DUSP1 is not involved in proteolytic processing of viral proteins during the maturation of MVA progeny particles.

To examine a later event in morphogenesis, we analyzed by immunofluorescence and confocal microscopy the distribution of the late viral membrane protein A27 (p14). As shown in Fig. 6C, in WT cells infected with MVA, a clear diffuse pattern of expression of A27 across the cytoplasm of the infected cell was observed indicating that A27 was expressed but it did not appear to be incorporated into virions. In contrast, in MVA-infected DUSP1 KO MEFs (Fig. 6C, upper right panel), A27 showed both a diffuse and a punctuated pattern (right-lower magnification) characteristic of the labeling of mature virions. Similar punctuated pattern of A27 was observed when DUSP1 expression was blocked in WT MEFs with siRNAs and infected with MVA (data not shown). These results suggest that DUSP1 might be involved in events that occur before the formation of mature virions.

**Figure 7:**
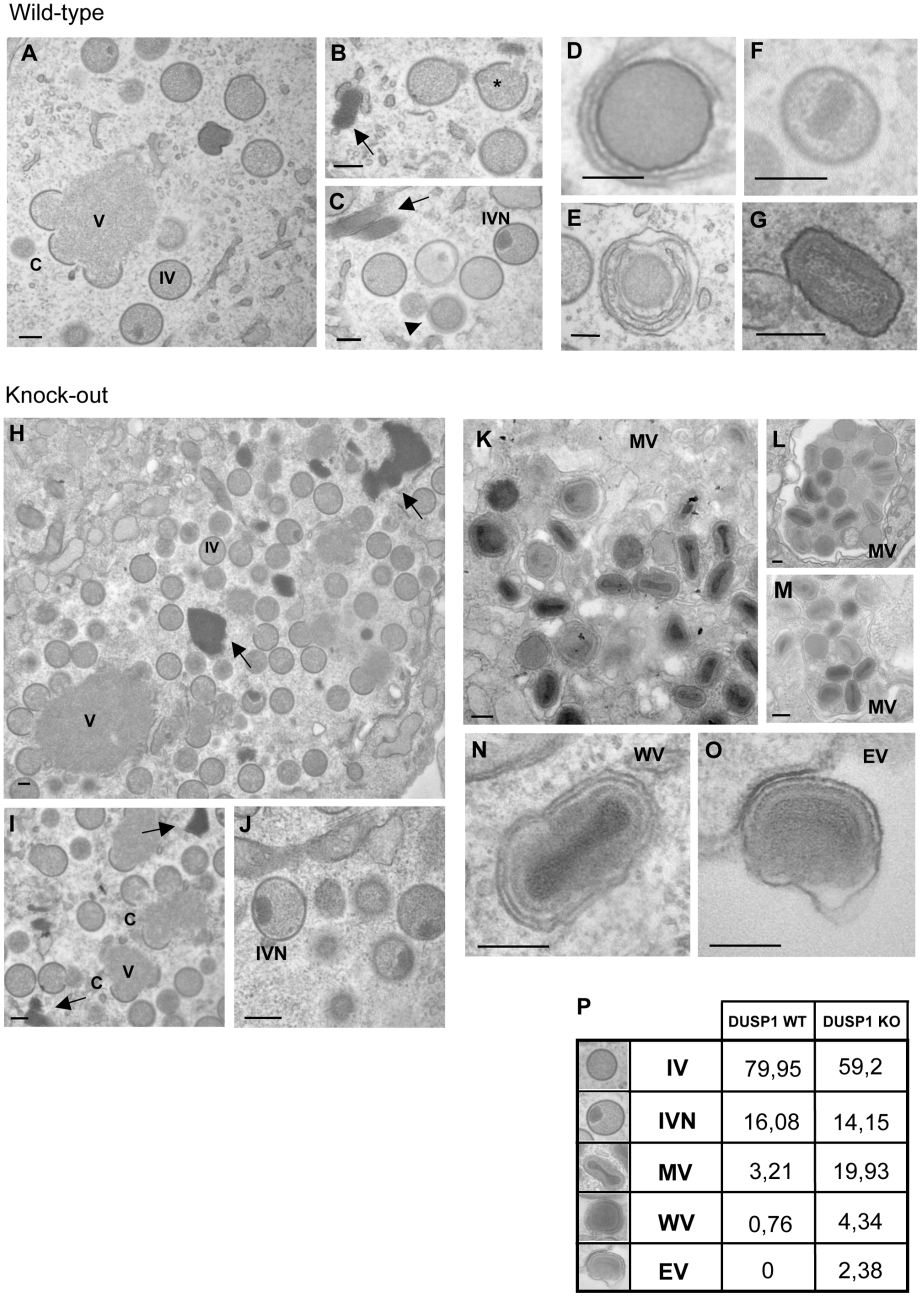
DUSP1 acts on the transition of immature and mature virion forms of MVA. DUSP1 WT and KO MEFs were infected with MVA at 5/cell, fixed at 16 hpi and processed by conventional embedding in a epoxy resin as described in Materials and Methods. **A**) Representative general overview of DUSP1 WT MEFs infected with MVA. Viroplasm foci (V) surrounded by crescents (C) and immature virions (IV) are also observed (left panel). **B–C**) DNA accumulations (arrows), sometimes delimited by membranes, and classical viral intermediate forms such as IV and IV with a nucleoid (IVN) are also observed. *Viral genome entering IV. **D, E**) Occasionally IVN-MV transition forms (arrowheads) are found surrounded by one or two membranes (onion-like particle). **F**) Rarely, aberrant virions with impaired nucleoid formation or **G**) mature virion (MV)-like particles with a non-condensed core are observed alone and scattered in the cytoplasm. **H–J**) Representative images of DUSP1 KO cells infected with MVA showing growing crescents (C) surrounding viroplasm foci (V). Also DNA accumulations (arrows), IVs and IVNs are observed. **K–M**) Representative images of MVs frequently found in the cytoplasm of KO infected cells, **L**) sometimes engulfed into a large vesicle. **N–O**) Representative images of WV and EV. Scale bars = 200 nm. **P**) Table representing the relative percentages of the different viral forms found in sections from 30 cells per cell line.

In order to identify the step of MVA morphogenesis affected by DUSP1, we performed electron microscopy analysis of DUSP1 WT and KO MEFs infected with MVA for 16 h at 5 PFU/cell. Figures 7A–C showed the most characteristic viral forms present in MVA-infected WT cells represented by small and medium viroplasm foci (V) surrounded by nascent crescents (C), immature virus (IV) and immature virus with nucleoid (IVN). In addition, there were also several transition forms from IVN to mature virus (MV) as electro-dense particles being wrapped by a membrane (Fig. 7D). These occasional intermediate forms have also been described during infection of HeLa cells with MVA [57]. Aberrant viral forms with multiple wrappings (onion-like particles, Fig. 7E) were also observed as well as virions with impaired nucleoid formation (Fig. 7F) and MV-like particles without a proper core condensation (Fig. 7G). In the case of DUSP1 KO cells infected with MVA, we detected all the classical viral forms, from viroplasm foci (V) with crescents, IV, IVN, MV, wrapped virus (WV) and enveloped virus (EV) (Figs. 7H–O). Groups of MV within a membrane were also observed (Fig. 7L). To further characterize MVA outcome in WT and KO infected cells, we quantified the percentages of the different viral forms by counting sections of 30 cells from each cell line (Fig. 7P). In MVA-infected WT cells, IV (80%) and IVN (16%) were the most abundant viral forms. The percentages of the different viral forms detected in MVA-infected DUSP1 KO cells were remarkably different with 59% of IV and almost the same amount (14%) of IVN compared to WT-infected cells, whereas MV accounted for 20%, a percentage equivalent to VACV outcome in a permissive cell line [56]. In addition, we were able to detect the presence of WV (4%) and EV (2%). The analysis by electron microscopy clearly showed that in DUSP1 KO cells, MVA is able to complete the morphogenesis process suggesting that DUSP1 is mainly acting at the stage of transition between IVN and MV.

### Activation of MAPKs contributes to the replication of MVA in DUSP1 KO MEFs

**Figure 8:**
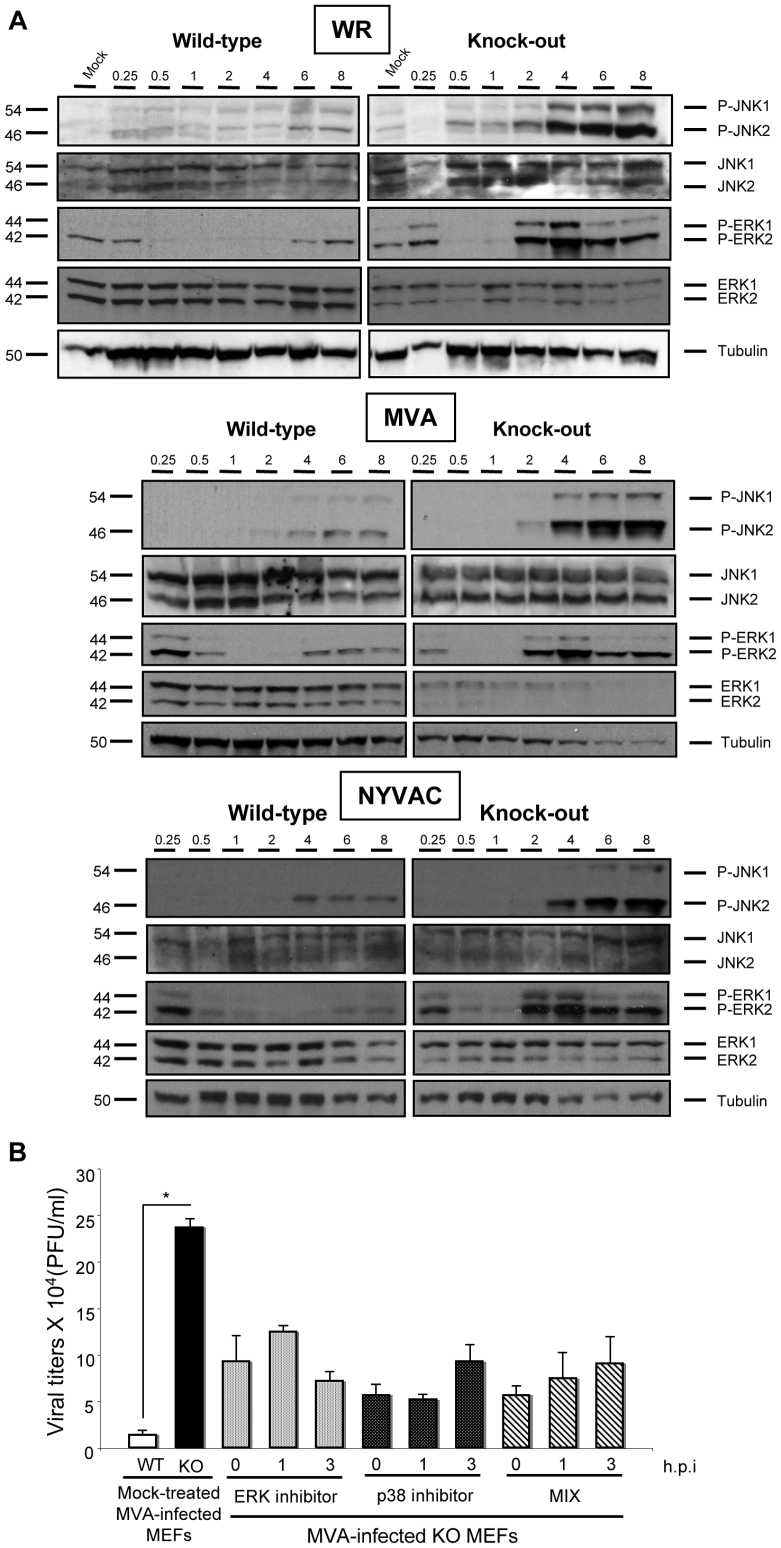
Activation of MAPKs contributes to the replication of MVA in DUSP1 KO MEFs. **A**) DUSP1 WT and KO cells were mock-infected or infected with either WR, MVA or NYVAC at 5 PFU/cell. Samples were collected at 0.25, 0.5. 1, 2, 4, 6 and 8 hours post-infection and 50 µg of total protein was analyzed by Western-blot using specific antibodies against P-ERK, ERK, P-JNK, JNK or tubulin. **B**) DUSP1 WT and KO cells were infected with MVA at 0.01 PFU/cell and were mock-treated or treated with either ERK inhibitor UO126 (10 µM/30 min) or p38MAPK inhibitor SB203580 (5 µM/1 h) at 0, 1 or 3 hpi. All samples were collected at 24 hpi and titrated in DF-1 cells by immunostaining plaque assay. Values shown are representative from two independent experiments performed.

Since DUSP1 is an important MAPK regulator and VACV infection is able to trigger or block specific molecules of this pathway such as ERK, p38MAPK and JNK [58], [59], [60], we next determined the effect of the absence of DUSP1 in the activation of these kinases during VACV infection. To address this issue, we infected DUSP1 WT and KO MEFs with WR, MVA or NYVAC at 5 PFU/cell and analyzed the expression of these proteins by Western-blot at 0.25, 0.5, 1, 2, 4, 6 and 8 hours post-infection. As shown in Fig. 8A, the three viruses triggered phosphorylation of ERK as early as 15 min post-infection. In the case of WT-infected MEFs (left columns), a modest phosphorylation of ERK was observed, while this activation was dramatically enhanced in the absence of DUSP1 (right columns). In KO-infected MEFs, ERK phosphorylation peaked between 2 and 4 hpi and this activation was maintained along the time course analyzed. We also observed that VACV infection preferentially phosphorylated ERK-2 in MEFs. A similar pattern of activation was observed in the case of JNK, although in this case we could not detect activation at early times post-infection. In KO MEFs infected with WR, MVA or NYVAC, JNK phosphorylation was markedly enhanced after 2 hours post-infection, while low levels of phosphorylation were detected in WT MEFs infected with any virus.

Altered activation of MAPKs during viral infection in the absence of DUSP1 might be the cause of the differences observed in VACV host range and replication. In order to analyze the specific contribution of each MAPK in the replication of MVA in DUSP1 KO-infected MEFs, we used MAPK inhibitors UO126 and SB203580 to block ERK and p38MAPK, respectively. The JNK inhibitor SP600125 could not be used because it has been recently described to exert anti-poxviral effects independent of its JNK inhibitory activity [61].

The MAPKs inhibitors were added to DUSP1 KO cells, previously infected with MVA, at 0, 1 or 3 hours post-infection and 24 hours later viral titers were determined. We observed about 2 to 5-fold decrease in MVA replication in DUSP1 KO-infected cells treated with the inhibitors in comparison with mock-treated KO-infected cells (Fig. 8B). From these findings, we conclude that activation of MAPKs by virus infection contributes to the replication of MVA in DUSP1 KO cells.

### DUSP1 KO mice are more susceptible to WR infection than WT mice

**Figure 9:**
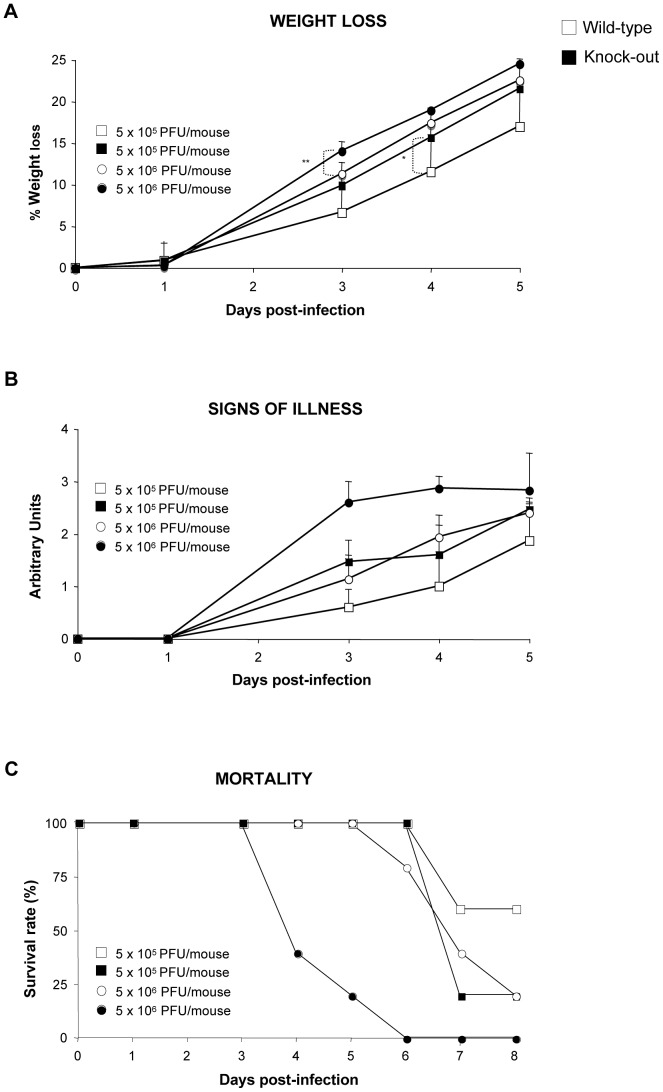
DUSP1 KO mice are more susceptible to WR infection than WT mice. DUSP1 WT or KO mice (n = 5) were intranasally (i.n) inoculated with either 5×10^5^ or 5×10^6^ PFU/mouse of WR. **A**) Mice were (individually) daily weighed and mean percentage weight loss of each group was compared with the weight immediately prior to infection. **B**) Signs of illness (lack of activity, ruffled fur) were daily evaluated using arbitrary units from 0 to 3 (healthy-ill). **C**) Survival rate after i.p. inoculation with WR. Different graphs show a representative example of two independent experiments performed. p<0.05 (*), p<0.005 (**) and p<0.001 (***).

The role of DUSP1 in bacterial and parasite diseases and clearance by the host has been well described [53]. However, little information has been reported regarding the involvement of DUSP1 in viral infections. To determine the function of DUSP1 during VACV infection, we inoculated DUSP1 WT and KO mice intranasally (i.n) with either 5×10^5^ or 5×10^6^ PFU/mouse of WR and monitored mice for different signs of disease like weight loss, illness (reduced mobility and ruffled fur) and mortality. DUSP1 KO mice experienced a significant (higher dose, day 3: *p*<0.005; low dose, day 4: *p*<0.05) more accelerated weight loss than their wild-type littermates during the course of the infection, with a reduction of 25% (high dose) or 21% (low dose) at day 5 post-infection (Fig. 9A). This susceptibility was also reflected by the worse disease prognosis evaluated daily during infection (Fig. 9B). Moreover, the onset of mortality of DUSP1 KO mice began as soon as 4 days post-infection, with the death of 3 out of 5 mice and reached the top at 6 days post-infection (Fig. 9C). In the case of DUSP1 WT mice it was not until day 6 when the first animal died and by the end of the experiment 20% were still alive (Fig. 9C). The viral titers in the lungs of infected animals at days 2 and 5 post-infection were higher in DUSP1 KO infected mice in comparison with WT mice at both times post-infection (Fig. S1). Together, these results demonstrate that DUSP1 deficiency leads to an enhanced susceptibility of mice to WR infection and this susceptibility could be related with an enhanced viral replication in the lungs.

In addition, we evaluated MVA and NYVAC infection of mice in the absence of DUSP1 administered by different routes of inoculation. By i.n route with a dose of 10^7^ PFU/mouse, neither DUSP1 WT nor KO mice infected with MVA or NYVAC displayed any sign of illness during the time period analyzed. Furthermore, we could not detect virus replication in the lungs of infected animals (data not shown). Next, we analyzed by a systemic i.p route with 2×10^7^ PFU/mouse the replication of MVA and NYVAC recombinants expressing luciferase. We collected spleens, lymph nodes, testis and peritoneal washes from infected animals at 4 and 16 hpi and evaluated luciferase activity as an index of viral replication (Fig. S2). We did not find differences in luciferase gene expression between WT and KO mice in the tissues analyzed, neither at 4 nor at 16 hpi. Finally, we skin-scarified groups of DUSP1 WT and KO mice with MVA (2×10^7^ PFU/mouse tail) and after 8 days post-infection we observed that lesions in the tails of KO mice were more severe than in their WT counterparts (Fig. S3); these lesions were not observed in media or PBS-scarified KO mice indicating that the inflammatory lesions were solely due to the virus inoculum. After virus titration of tail tissue samples from infected animals, we did not find evidence of MVA replication (data not shown), suggesting that the more severe lesions observed in DUSP1 KO mice were related to a host inflammatory response triggered by MVA infection in the absence of DUSP1.

### DUSP1 is involved in the regulation of innate and adaptive immune responses during VACV infection

DUSP1 is known to be an essential regulator of the host inflammatory response that controls the production of several cytokines following different stimuli [40]. It has been well established that the enhanced inflammatory response, triggered by the hyper-phosphorylation of MAPK in the absence of DUSP1, can even kill the host [62]. At present, little is known about the role of DUSP1 in host immune responses during viral infections. To further analyze this effect, we first focused on innate immune responses triggered after systemic (i.p) infection of DUSP1 WT and KO mice with 10^7^ PFU/mouse of WR or 2×10^7^ PFU/mouse of MVA or NYVAC. We determined production of IL-6, TNF-α, IL-1β and IL-10 at 3 and 24 hpi in serum from infected mice. These cytokines were selected since they are the most commonly induced cytokines triggered by other stimuli in the absence of DUSP1. As shown in Fig. S4, most of these cytokines were not or poorly induced in WT and KO mice infected with WR. However, infection with MVA or NYVAC induced the expression of IL-6, TNF-α and IL-10, being this induction higher in KO mice. Differences in levels and in temporal induction of these cytokines were found between infections with MVA versus NYVAC in DUSP1 KO mice, an expected finding since these two viruses differ in the number of immunomodulatory genes present in their genomes [6].

**Figure 10:**
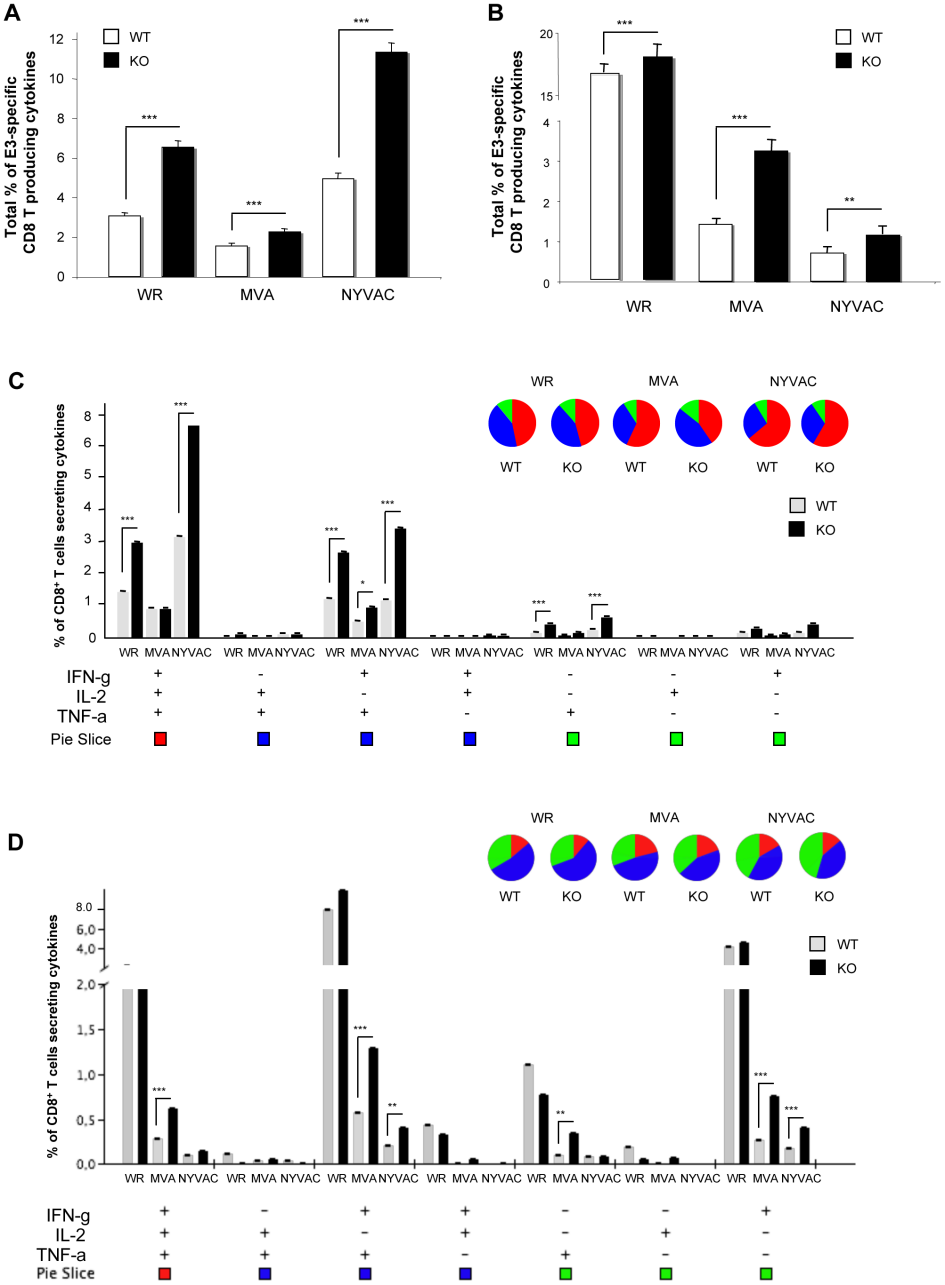
DUSP1 is involved in the regulation of adaptive immune response during VACV infection. **A**) Magnitude of E3-specific CD8 T cell response after intraperitoneal (i.p.) inoculation with either WR (10^7^ PFU/mouse), MVA (2×10^7^ PFU/mouse) or NYVAC (2×10^7^ PFU/mouse). The values represent the sum of the percentages of CD8^+^ T cells secreting IL-2 and/or TNF-α and/or IFN-γ. CD8^+^ T cell responses were measured 10 days after inoculation by ICS assay following stimulation of splenocytes with E3 peptide. All data are background subtracted. **B**) Magnitude of E3-specific CD8 T cell response after intranasal (i.n) inoculation with either WR (10^5^ PFU/mouse), MVA (5×10^7^ PFU/mouse) or NYVAC (5×10^7^ PFU/mouse). **C**) Functional profile of E3-specific CD8 T cell responses after i.p inoculation as in A). All the possible combinations of the responses are shown on the *x* axis and the percentage of the functionally distinct cell populations within the total CD8 T cell populations are shown on the *y* axis. Responses are grouped and color-coded on the basis of the number of functions. **D**) Functional profile of E3-specific CD8^+^ T cell responses after i.n. as in B). Results are represented as in C). Graphs show a representative example of three independent experiments performed. p<0.05 (*), p<0.005 (**) and p<0.001 (***).

Next, we characterized T cell responses after VACV infection, analyzing the pattern of cytokine secretion by intracellular cytokine staining (ICS) from splenocytes stimulated with the immunodominant VACV E3 peptide. DUSP1 WT and KO mice were i.p. inoculated with WR (10^7^ PFU/mouse), MVA or NYVAC (2×10^7^ PFU/mouse) and animals sacrificed after 10 days post-infection. Alternatively, we inoculated mice by i.n. route with WR (10^5^ PFU/mouse), MVA or NYVAC (5×10^7^ PFU/mouse). After stimulating splenocytes from infected animals with the VACV-E3 peptide, we analyzed by ICS the production of IFN-γ, IL-2 and TNF-α. As shown in Fig. 10A, the magnitude of E3-specific CD8 T cell response after i.p. inoculation, determined as the percentages of IFN-γ and/or IL2 and/or TNF-α CD8 secreting cells, was significantly higher (*p*<0.001) in DUSP1 KO infected mice with the three viruses, although it was more remarkable in infections with WR and NYVAC. The antigen-specific immune response elicited by the three viruses by i.p. inoculation was highly polyfunctional and mainly represented by triple and double positive T cells for TNF-α and IFN-γ with significant differences (*p*<0.001) in the magnitude of those populations between DUSP1 WT and KO mice infected with WR or NYVAC but not with MVA (Fig. 10C). Infections by the i.n. route revealed also significant differences (*p*<0.001) in the magnitude (Fig. 10B) and polyfunctionality (Fig. 10D) of the E3-specific CD8 T cell responses between the three viruses, particularly for DUSP1 KO infected animals. In this case, the polyfunctional profile was represented by double positive CD8 T cells for IFN-γ and TNF-α and single positive CD8 T cells for IFN-γ (Fig. 10D).

The findings of Figs. 10 and S4 reveal that DUSP1 is involved in the regulation of innate and adaptive immune responses during a poxvirus infection, as shown by differential behavior of WR, MVA and NYVAC regarding cytokine production and by the higher percentage of cytokine-secreting CD8^+^ T cells induced in DUSP1 KO mice in comparison with WT animals.

## Discussion

At present, little is known about the role of DUSP1 in viral infections. Our study has addressed this issue by using the VACV system in both cultured murine cells and in mice lacking DUSP1. In particular, we have shown the importance of DUSP1 in VACV infection, from viral replication and host range to host immune responses. In cultured cells, we have demonstrated that VACV specifically upregulates DUSP1 during infection and this induction is dependent on early viral protein synthesis. The higher upregulation of DUSP1 mRNA in cells infected in the presence of CHX suggests that viral RNAs released by viral cores (as CHX blocks uncoating but allows RNA synthesis) are responsible for DUSP1 mRNA induction. Moreover, VACV infection triggers DUSP1 phosphorylation through activation of ERK, discarding the possibility of phosphorylation by a viral kinase and demonstrating that VACV not only triggers DUSP1 upregulation but also DUSP1 protein stabilization. These results are consistent with previous reports, which showed that VACV promotes ERK activation allowing an efficient viral replication in the infected cells and successful viral progeny release [17], [59], [7].

Of relevance, we have observed an increase of VACV replication in DUSP1 KO immortalized MEFs compared to WT cells. Specifically, we have detected an increase in WR viral titers but more interestingly, in MVA viral titers. In addition, we have further confirmed this enhanced WR and MVA replication in DUSP1 KO MEFs using specific siRNAs against DUSP1 in WR or MVA infection in cells from different origin such as HeLa cells, and also restoring DUSP1 expression in DUSP1 KO cells. Permissiveness to poxvirus replication is mostly dependent on intracellular events after entering the cells, such as several host protein kinase cascades that can mediate subsequent viral replication events rather than on cell surface receptors. For example, VACV can even enter non-permissive cell lines as insect cells [63]. In addition, although many poxviruses show strict species specificities, these can vary markedly such that cells derived from species that are not considered permissive hosts can sometimes be productively infected *in vitro* [64]. Phosphorylation is an important event that numerous viruses use during their replication process [65], [66]. In the context of this study, the absence of DUSP1 led to a change in the host range of MVA. This result might be related with the observed changes in the pattern of MAPK activation during MVA infection in the absence of DUSP1. Therefore, we analyzed whether the inactivation of ERK and p38MAPK by specific inhibitors had an implication on MVA replication in DUSP1 KO cells. We have observed a reduction in MVA viral titers in comparison with untreated DUSP1 KO cells infected with MVA, suggesting that these MAPKs are directly involved in the phenotype observed in the absence of DUSP1. There are several possible explanations for these results. First, MAPKs may be acting over cellular ligands to promote their stabilization, activation or changes in their subcellular localization. Thus, the MAPK-mediated hyper-phosphorylation in the absence of DUSP1 during MVA infection may be miss-regulating these processes in a manner that MVA is able to replicate in murine cells. Alternatively, this phenotype may be due to MAPK action over viral phosphoproteins. VACV has been widely reported to use phosphorylation as a mechanism to regulate the activity of several viral proteins [67]. Most of them are phosphorylated by VACV-encoded kinases B1 and F10 [14], [68] but others are phosphorylated by cellular kinases [67]. Many of these phosphoproteins have an important role during VACV morphogenesis [69], [70], [71], [72]. Then, the phosphorylation status of viral structural proteins may be affected by the absence of DUSP1 during MVA infection and this may have an effect on virus morphogenesis.

By Western-blot and confocal microscopy on VACV-infected DUSP1 WT and KO cells, we did not observe changes in subcellular viral protein localization nor in proteolytic processing of major core and viral structural proteins, like L4, A16 and A17. This is consistent with previous studies that showed the same phenotype when MVA infects non-permissive cell lines such as HeLa [73]. However, when we analyzed the expression of the late membrane protein A27, we found that A27 was incorporated in virions in DUSP1 KO cells infected with MVA but its expression was mostly diffuse across the cytoplasm in the case of DUSP1 WT infected cells. These results point to an implication of DUSP1 in the events that occur during the transition from IVN to MV. The majority of the mutations that affect virion morphogenesis are involved in this complex process [67]. Whether the role of DUSP1 in VACV morphogenesis is strictly dependent of viral protein assembly or it is associated with other cellular processes remains to be determined.

In fact, electron microscopy data of the viral morphogenesis steps of DUSP1 WT and KO cells infected with MVA revealed that MVA is able to complete its viral cycle in the absence of DUSP1, further supporting the findings of time course analysis and confocal microscopy. While all different viral forms (crescents, IV, IVN, MV, WV and EV) were produced in DUSP1 KO infected cells, similarly as it occurs during a productive VACV infection, in WT MEFS the mature viral forms MV and EV were greatly impaired, according to the phenotype of a non-permissive cell line for MVA infection [56]. Comparative data of the different viral forms shows quantitatively that the main block in WT cells occurs at the transition between IVN and MV.

We have also defined the influence of DUSP1 during VACV replication *in vivo* in the mouse model. The increased weight loss, higher death rate and worse disease prognosis after i.n. inoculation with WR revealed that the absence of DUSP1 leads to an increase susceptibility of infected mice. One possible cause for this susceptibility could be an exacerbated pro-inflammatory cytokine production but we did not find detectable levels of these cytokines in lungs from DUSP1 WT or KO mice infected with WR. However, we observed differences between cytokine levels in serum from WT and KO mice infected with the attenuated viruses MVA or NYVAC. Hence, the main possible explanation for the susceptibility of DUSP1 KO mice to WR infection may be the higher viral titers found in the lungs of DUSP1 KO mice infected with WR in comparison with WT mice.

Previous studies have shown the pivotal role of the modulation of MAPK in host immune responses [74], [75]. Moreover, in recent years, there has been an increasing amount of literature on the role of DUSP1 in the inflammatory response against different pathogens [40], [62], [76], [77]. Taking into account these data, we considered interesting to address the involvement of DUSP1 in innate and adaptive immune responses after VACV infection. Measurement of cytokine production in the serum of infected mice revealed that DUSP1 KO mice infected with NYVAC experienced enhanced pro-inflammatory cytokine release as soon as 3 hpi, whereas in DUSP1 KO mice infected with MVA this enhancement is observed at 24 hpi. These differences in the kinetics of cytokine production may be explained by the fact that these two VACV attenuated viruses lack different immunomodulatory genes and, consequently, their immunogenicity profile affects MAPK signaling pathway in a distinct manner.

In adaptive immune responses, MAPKs also serve as critical regulators in the clonal expansion of effector T and B lymphocytes through modulation of cytokine production, cell proliferation and survival [78]. As a key MAPK modulator, DUSP1 may play an important role during antiviral adaptive immune responses. Therefore, we characterized the adaptive immune responses by ICS after i.n. and i.p. inoculation with VACV. The results indicate that even though there were differences in the magnitude of CD8^+^ T cell responses triggered by WR, MVA or NYVAC, viral-specific CD8 T cell responses were higher in DUSP1 KO mice infected than in WT animals, clearly suggesting that DUSP1 modulates adaptive immune responses during VACV infection. As WR had an enhanced replication in lungs from DUSP1 KO mice, the amount of viral antigen produced was higher and longer expressed in the organism with this fully competent replicating virus in DUSP1 KO mice than in DUSP1 WT mice. In the case of the attenuated MVA and NYVAC, the increased CD8^+^ T cell response observed in DUSP1 KO mice might be related to the different innate immune responses triggered by these viruses due to the absence of different immunomodulatory genes in their genomes [6], [79].

The adaptive immune responses triggered can be shaped by the elicited innate immune response [80]. For instance, dendritic cells (DCs) are one of the first lines of defense against pathogens and the most important cells to bridge innate and adaptive immunity [81]. In this sense, it has been recently reported that DUSP1 has an essential role in the integration of DC-signals and T cell responses, coordinating protective immunity and immunopathology against bacteria and fungi [82]. Thus, it is also possible that the absence of DUSP1 in innate immune cells is responsible for the variations in T cell responses observed after VACV infection.

In summary, this is the first report demonstrating the role of DUSP1 during VACV infection. We can conclude that DUSP1 expression is specifically upregulated and phosphorylated during VACV infection and that the activation of ERK by VACV is necessary to promote DUSP1 phosphorylation. More interestingly, host restriction of MVA replication in murine cells is overcome when DUSP1 is absent and activation of MAPKs by virus infection contributes to this enhanced replication. We have demonstrated that DUSP1 is mainly acting at the transition between IVN and MV. In addition, we have shown that DUSP1 deficiency results in an enhanced susceptibility of mice to WR infection and this susceptibility could be related with an enhanced viral replication in the lungs; meanwhile the more severe skin lesions observed in DUSP1 KO mice were likely due to a host inflammatory response triggered by MVA. Finally, we have demonstrated that DUSP1 is involved in the modulation of innate and adaptive immune responses during VACV infection. Overall, DUSP1 acts as an antiviral host defense factor against a poxvirus infection.

## Supporting Information

Figure S1 **DUSP1 modulates WR replication in the mouse model.** Mice were intranasally (i.n) inoculated with WR-luc (5×10^6^ PFU/mouse) and luciferase gene expression and viral titers in the lungs from DUSP1 WT and KO-infected mice were evaluated at days 2 and 5 post-infection (n = 4/day/group), by luciferase activity and virus plaque assays as described under Materials and Methods. Graph shows a representative example of three independent experiments performed.
(PDF)

Figure S2 **MVA and NYVAC replication during intraperitoneal infection in DUSP1 WT and KO mice.** DUSP1 WT and KO mice (n = 4) were i.p inoculated with 2×10^7^ PFU/mouse with MVA-luc (**A**) or NYVAC-luc (**B**). Mice tissue samples were collected at 4 and 16 hpi. MVA replication in spleen, lymph nodes testis and peritoneal washes, was measured as Luciferase activity and represented as Units/mg tissue.
(PDF)

Figure S3 **DUSP1 KO mice develop tail lesions after MVA skin scarification.** Mice (n = 4) were skin-scarified in the tail with MVA (10^7^ PFU/mouse) and were daily monitored for lesion appearance. Representative images from DUSP1 WT and KO mice at days 8 and 11 post-infection are shown. Three independent experiments were performed.
(TIF)

Figure S4 **Enhanced innate immune response during VACV infection in the absence of DUSP1.** Mice (n = 3) were i.p inoculated with either WR (1×10^7^ PFU/mouse), MVA (2×10^7^ PFU/mouse) or NYVAC (2×10^7^ PFU/mouse). IL-6, TNF-α, IL-1β and IL-10 were analyzed by LUMINEX technology from serum extracted at the indicated times post-infection.
(PDF)
