# Supplementary figures and images for: Involvement of the Cellular Phosphatase DUSP1 in Vaccinia Virus Infection
Source: PLoS Pathog. 2013 Nov 14;9(11):e1003719. doi: 10.1371/journal.ppat.1003719 (PMC3828168; doi:10.1371/journal.ppat.1003719)

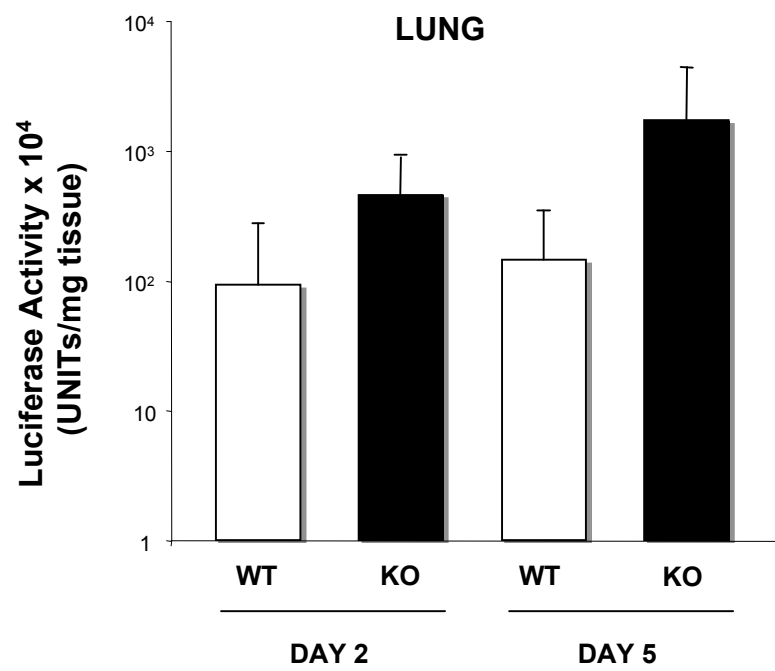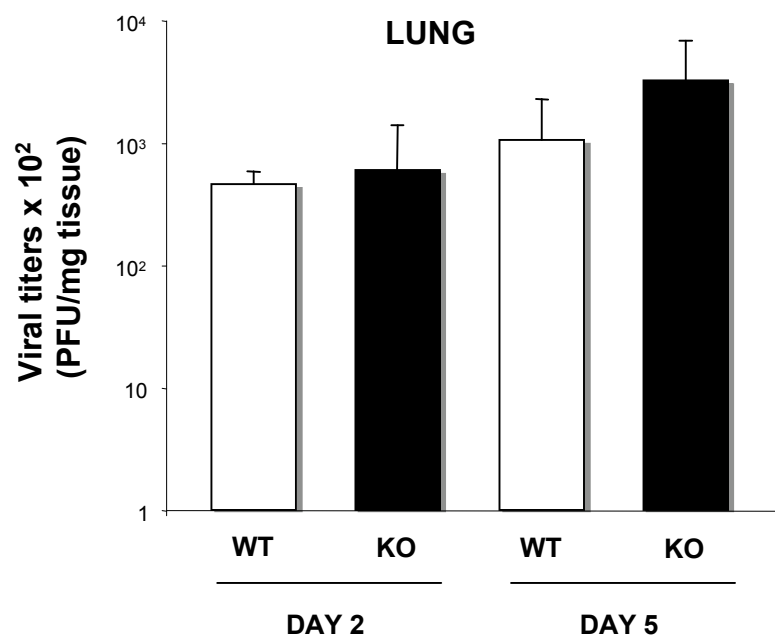

Supplement: Figure S1 — DUSP1 modulates WR replication in the mouse model. Mice were intranasally (i.n) inoculated with WR-luc (5×10^6^ PFU/mouse) and luciferase gene expression and viral titers in the lungs from DUSP1 WT and KO-infected mice were evaluated at days 2 and 5 post-infection (n = 4/day/group), by luciferase activity and virus plaque assays as described under Materials and Methods. Graph shows a representative example of three independent experiments performed. (PDF) [file ppat.1003719.s001.pdf]

**A****MVA-LUC**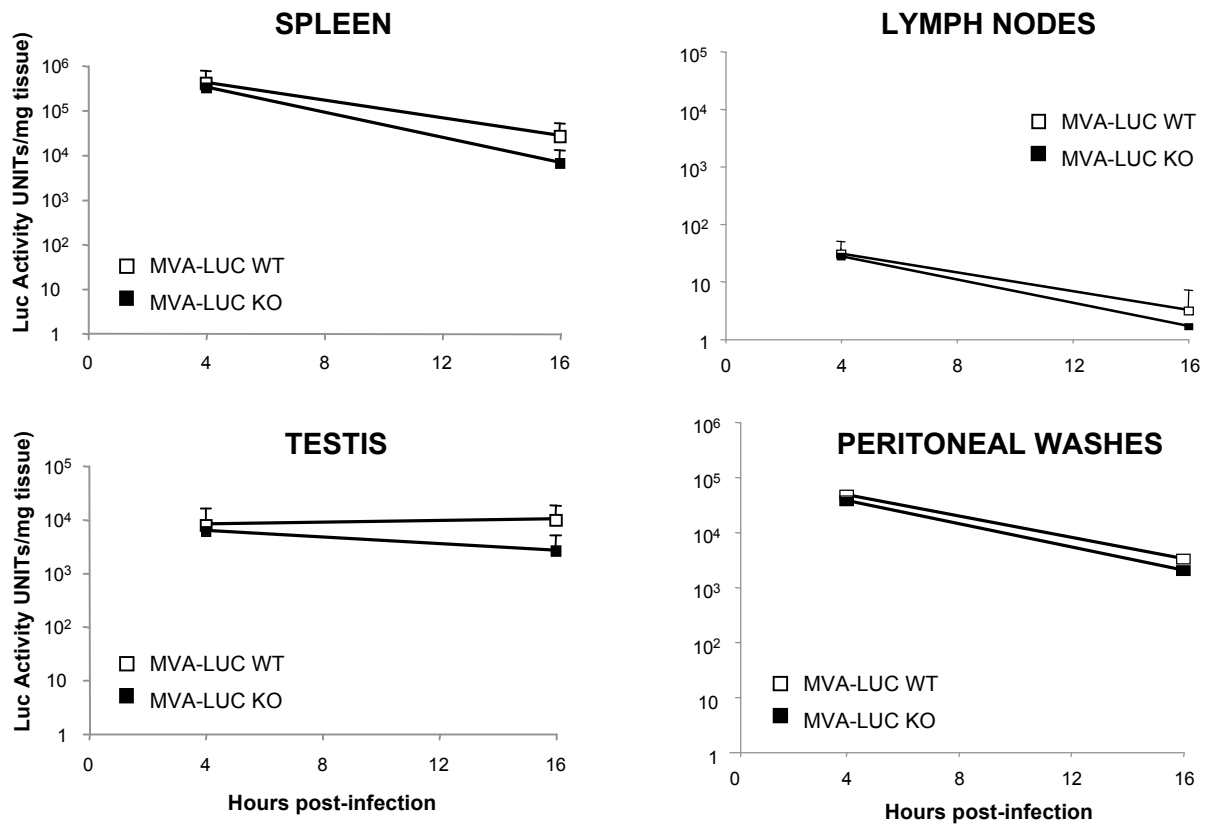**B****NYVAC-LUC**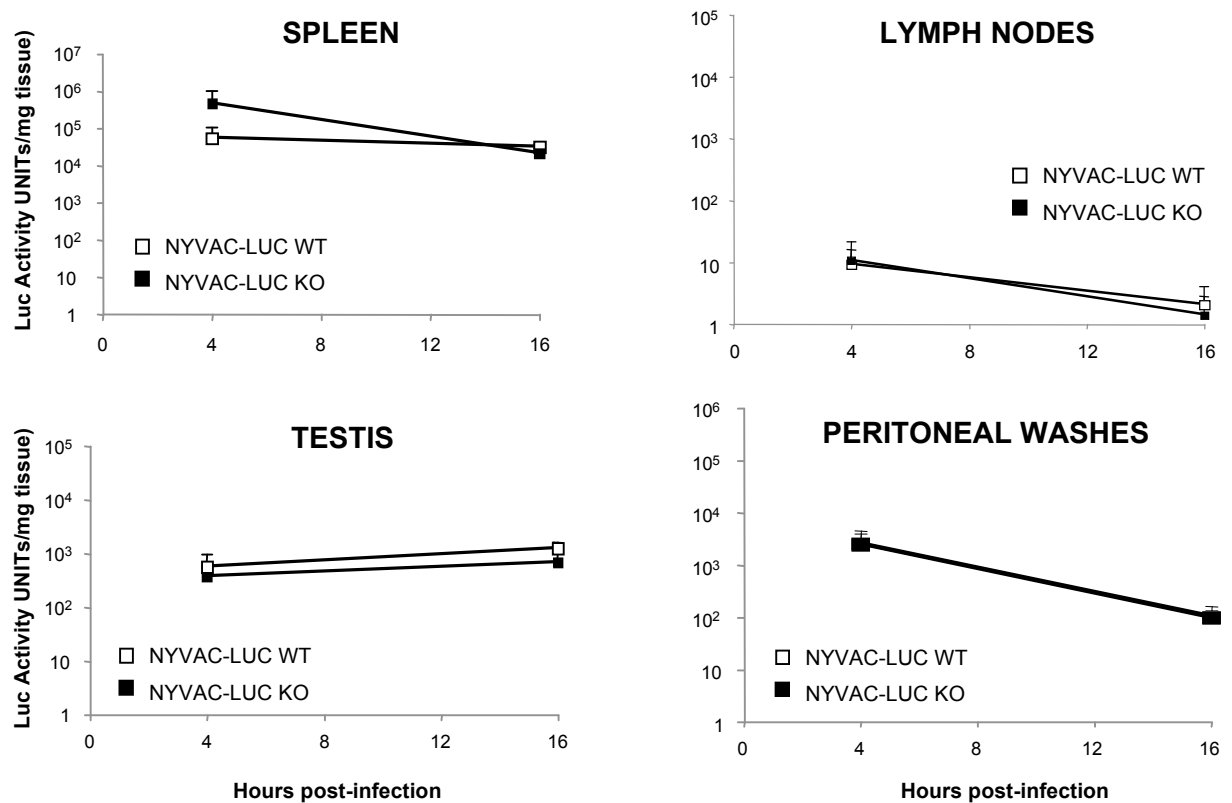

Supplement: Figure S2 — MVA and NYVAC replication during intraperitoneal infection in DUSP1 WT and KO mice. DUSP1 WT and KO mice (n = 4) were i.p inoculated with 2×10^7^ PFU/mouse with MVA-luc (A) or NYVAC-luc (B). Mice tissue samples were collected at 4 and 16 hpi. MVA replication in spleen, lymph nodes testis and peritoneal washes, was measured as Luciferase activity and represented as Units/mg tissue. (PDF) [file ppat.1003719.s002.pdf]

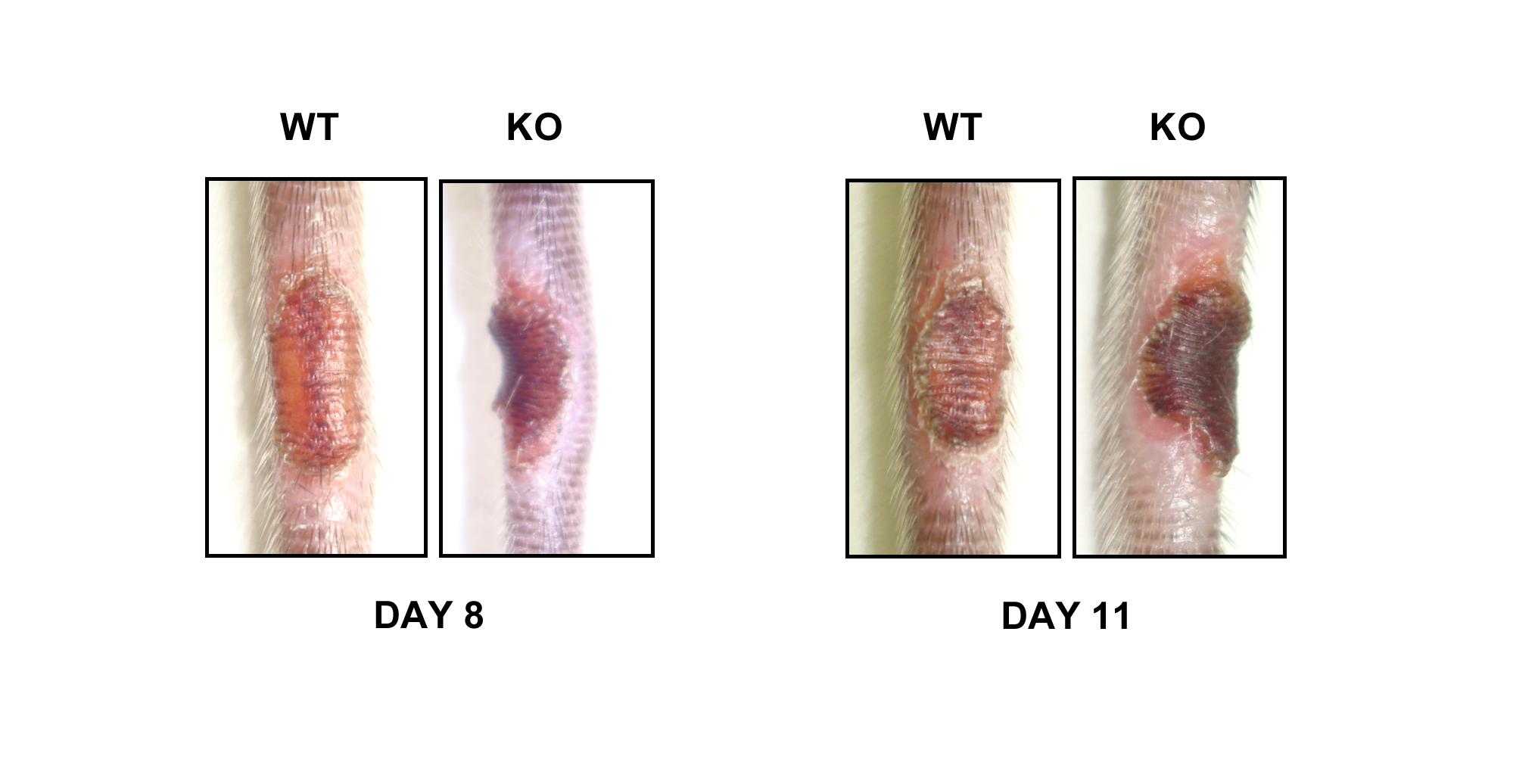

Supplement: Figure S3 — DUSP1 KO mice develop tail lesions after MVA skin scarification. Mice (n = 4) were skin-scarified in the tail with MVA (107 PFU/mouse) and were daily monitored for lesion appearance. Representative images from DUSP1 WT and KO mice at days 8 and 11 post-infection are shown. Three independent experiments were performed. (TIF) [file ppat.1003719.s003.tif]

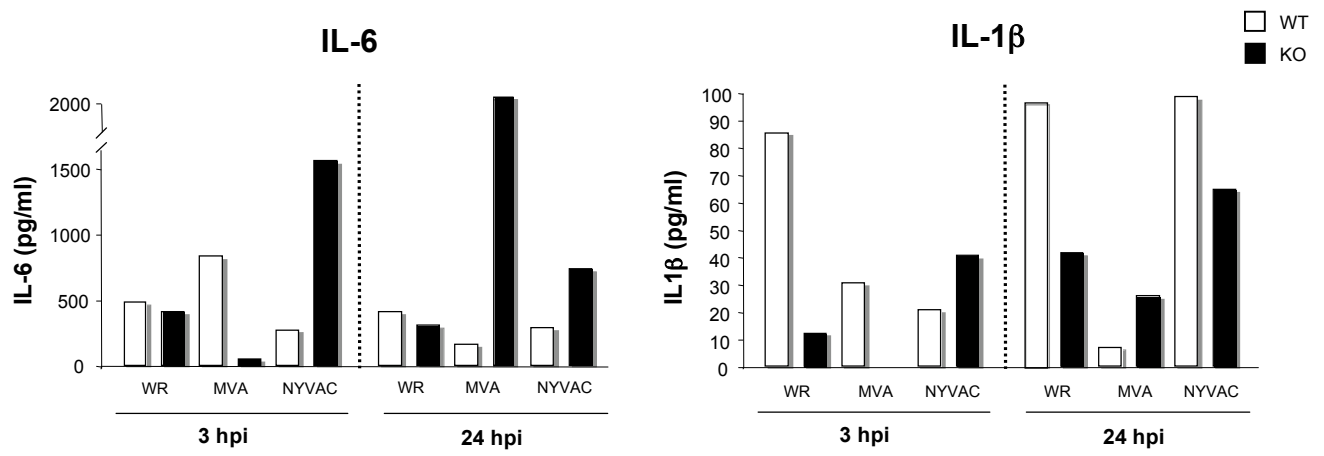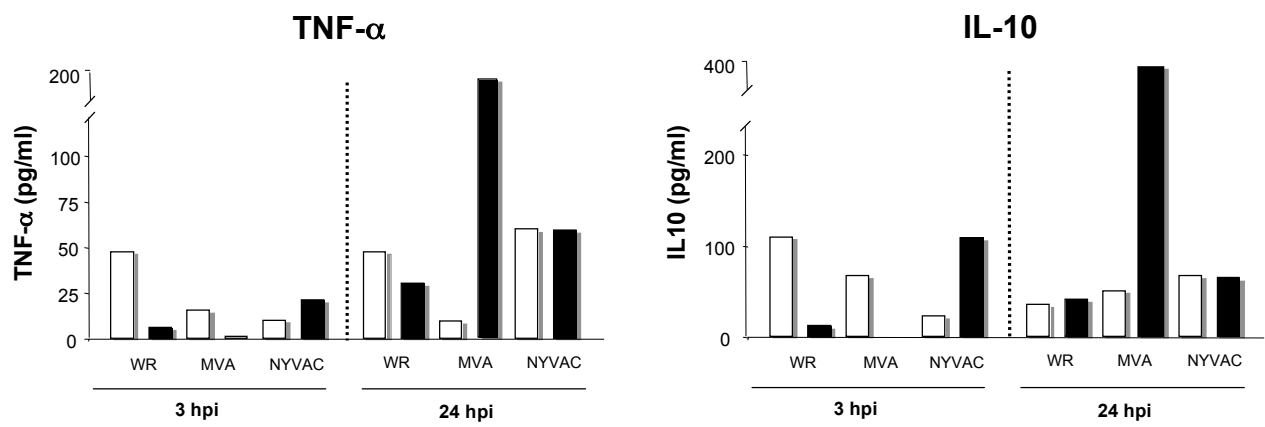

Supplement: Figure S4 — Enhanced innate immune response during VACV infection in the absence of DUSP1. Mice (n = 3) were i.p inoculated with either WR (1×10^7^ PFU/mouse), MVA (2×10^7^ PFU/mouse) or NYVAC (2×10^7^ PFU/mouse). IL-6, TNF-α, IL-1β and IL-10 were analyzed by LUMINEX technology from serum extracted at the indicated times post-infection. (PDF) [file ppat.1003719.s004.pdf]
